# The Effects of Soy Protein–Rich Meals on Muscle Health of Older Adults Are Linked to Gut Microbiome Modifications

**DOI:** 10.1002/jcsm.70212

**Published:** 2026-01-25

**Authors:** Xiaorong Wu, Kevin Junliang Lim, Yiwei Ma, Jie Gu, Yuanrong Jiang, Liying Zhu, Yanqiu Chen, Jianqing Sun

**Affiliations:** ^1^ WIL@NUS Corporate Laboratory National University of Singapore, Centre for Translational Medicine Singapore; ^2^ Wilmar (Shanghai) Biotechnology Research & Development Center Co. Ltd Shanghai China; ^3^ Institute of Food Science, Zhejiang Academy of Agricultural Sciences Hangzhou Zhejiang China; ^4^ Clinical Nutrition Center Fudan University Affiliated to Hua Dong Hospital Shanghai China; ^5^ Shanghai Elderly Nutrition and Health Quality Control Center Shanghai China

**Keywords:** gut microbiome, muscle function, older adults, sarcopenia, short‐chain fatty acids, soy protein

## Abstract

**Background:**

Sarcopenia is characterized by accelerated muscle mass and function loss in older adults. The role of nutritional interventions in sarcopenia is uncertain. This study investigates whether a soy protein–rich diet can enhance muscle health in older adults via gut microbiota changes.

**Methods:**

A 12‐week randomized controlled trial was conducted with 84 older adults from a long‐term care facility. Participants in the intervention group consumed three daily meals containing 10 g of soy protein (totalling 30 g/day), while the control group maintained their usual diets. Faecal samples from 53 participants were collected at Weeks 0, 6 and 12. We assessed changes in muscle function, gut microbiota composition and faecal short‐chain fatty acids (SCFA).

**Results:**

The intervention group showed preserved calf circumference, while the control group experienced a decrease (W12‐W0: Intervention, 0.56 ± 0.22 cm; Control, −0.91 ± 0.26 cm, *p*_(interaction)_ < 0.001). Metagenomic analysis revealed significant alterations in gut microbiota among intervention participants who showed improvement in muscle performance parameters. The intervention increased SCFA‐producing bacteria ( *Roseburia faecis* , Intervention: 0.42 ± 0.21%, Control: −0.06 ± 0.16, *p*_(interaction)_ < 0.05; *Agathobaculum butyriciproducens* , Intervention: 0.02 ± 0.007%, *p*_(time)_ < 0.01, Control: −0.04 ± 0.01) and decreased species associated with poorer muscle outcomes ( *Alistipes putredinis* , Intervention: −0.88 ± 0.40%, Control: 0.62 ± 0.63, *p*_(interaction)_ < 0.05; *Eubacterium_sp_CAG_38*, Intervention: −0.64 ± 0.28%, Control: 0.10 ± 0.22, *p*_(interaction)_ < 0.05). Functional pathway analysis showed enrichment of anaerobic amino acid degradation pathways and vitamin biosynthesis, with depletion of inflammatory pathways, particularly lipopolysaccharide biosynthesis. Microbiome phenotype prediction revealed a decrease in aerobic bacteria abundance in the intervention group (W12‐W0, Intervention: −0.004 ± 0.002; Control: 0.001 ± 0.001, *p*_(interaction)_ < 0.05). Interaction (group × time) for SCFA was not statistically significant; within‐group increases at Week 6 were observed in only the intervention group (butyric acid, Intervention: 0.74 ± 0.34 mg/g, *p*_(time)_ < 0.05, Control: 0.12 ± 0.43 mg/g; isobutyric acid, Intervention: 0.14 ± 0.08 mg/g, *p*_(time)_ < 0.05, Control: 0.08 ± 0.10 mg/g; isovaleric acid, Intervention: 0.27 ± 0.14 mg/g, *p*_(time)_ < 0.05; Control: 0.16 ± 0.20 mg/g), with partial reversal by Week 12. These changes, positively correlated with improved muscle function parameters, suggest intervention benefits on gut health and muscle function.

**Conclusion:**

A soy protein–rich intervention improved muscle health in older adults through beneficial gut microbiota. These findings support the gut–muscle axis hypothesis and suggest dietary soy protein may alleviate sarcopenia by promoting a healthier gut microbiome.

## 1 Introduction

Sarcopenia has been defined as a progressive and generalized skeletal muscle disorder that involves the accelerated loss of muscle mass and function. Sarcopenia is associated with increased adverse outcomes including falls, functional decline, frailty and mortality [1], all of which substantially affect the quality of life of affected individuals and increase their medical burden. Although there are many causes of sarcopenia, including nutritional, physical inactivity, disease, and iatrogenic, previous studies have also shown that age‐related sarcopenia is the primary [2]. China's seventh national census revealed that the proportion of people aged 60 and above accounted for 18.7% of the total Chinese population in 2020. This number is even higher for Shanghai, affecting 23.4% of the population [3]. Consequently, the prevention and treatment of sarcopenia is becoming increasingly important and urgent as the Chinese population ages.

Evidence‐based clinical practice guidelines were published in 2018 and provide strong recommendations for physical activity as the primary treatment of sarcopenia [4]. The evidence for the benefits of resistance exercise in improving individual skeletal muscle strength and mass is compelling [5, 6]. However, the role of a nutritional intervention in treating sarcopenia is much less clear and inconsistent [4]. Some studies have suggested the benefits of adopting healthier dietary patterns, such as adequate protein intake, vitamin D, antioxidant nutrients and long‐chain polyunsaturated fatty acids [7]. The most recent consensus recommendation is to increase the intake of protein in the elderly population [8, 9]. Two previous studies showed that in the specific context of sarcopenia with malnutrition, high‐protein oral supplements may be effective in improving some outcomes of sarcopenia [10, 11], even though the results were not too significant. However, another intervention trial done in nonsarcopenic patients with reduced mobility comparing the effects of normal versus increased protein intake on mobility showed no differences between these interventions [12].

Ageing disturbs the homoeostasis of skeletal muscle, which requires balance between hypertrophy and regeneration through complex and not yet fully understood mechanisms and pathways. Clinical research has shown that ageing of muscle tissue is characterized by loss of mass and function, degeneration of the neuromuscular junction (NMJ) and reduced regenerative capacity [13]. Ageing appears to result in an imbalance between protein anabolism and catabolism in the elderly, leading to an overall loss of skeletal muscle [14]. Similarly, it has been reported that there are significant alterations in the proportion and composition of the gut microbiota in older adults, leading to reduced microbiota diversity, as well as an increase of enteropathogens that may lead to chronic inflammation [15, 16, 17, 18]. The gut microbiota is of vital importance to life and health, and a growing body of evidence linking the gut microbiota and skeletal muscle mass has led to the concept of the gut–muscle axis. A study showed that dysbiosis of the gut microbiome caused by continuous antibiotic treatment was associated with blunted fibre type‐specific hypertrophy in the soleus and plantaris muscles in response to progressive weighted wheel running (PoWeR), and with impaired PoWeR‐induced fibre‐type shift and myonuclei accretion in the plantaris muscle [19]. Another study showed that gut microbiota affects skeletal muscle health in menopausal women through the production of butyrate [20]. Butyric acid also affects skeletal muscle health by mitigating antibiotic‐induced satellite cell (SC) activation via monocarboxylate transporter 1 (MCT1) to enhance SCs homeostasis and function during skeletal muscle ageing [21]. In addition, alteration of gut microbiota may also lead to skeletal muscle atrophy via a bile acid‐farnesoid X receptor (FXR) pathway [22]. These studies are evidence of the inextricable link between gut microbiota and its metabolites and skeletal muscle health.

Soybean is a traditional Chinese health food that has long been consumed in China. Various soybean‐based products have been developed, including tofu, soy milk, soy sauce and soybean paste, all of which are affordable, widely available and well‐accepted by people. Furthermore, soy protein is a complete protein in that it contains all essential amino acids [23], so soybeans and their products can serve as excellent alternative sources of protein, especially given the common occurrence of lactose intolerance among the Chinese population [24]. Current research into soy protein's effects on improving muscle health in older adults in care is limited. In our randomized controlled trial, participants received a soy protein–enriched dietary intervention consisting of daily supplementation with soy protein for 12 weeks. Earlier findings from this trial demonstrated the impact of a soy protein–enriched dietary intervention on muscle health, body composition, anthropometric measurements and physical performance in elderly residents (*n* = 84) of a long‐term care facility [25]. Here, we focused on a subset of participants (*n* = 53) to investigate alterations in gut microbiota composition and their relationship with improvements in skeletal muscle health.

## 2 Methods

### 2.1 Research Design

A 12‐week single‐centre randomized controlled trial with a parallel‐group and open‐label design was conducted. The present study is registered in the China Clinical Trials Registry (ChiCTR2000039301) and was approved by the Ethical Review Committee of Huadong Hospital, which is affiliated with Fudan University (2020K178). The trial protocol is available from the corresponding author upon reasonable request. All participants provided written informed consent for their participation. The data used in the present study were collected between December 2020 and February 2022 in Shanghai. The study was conducted at the XiJiao Union Retirement Center in Changning District, Shanghai.

### 2.2 Participants

Older adults living in a long‐term care facility (i.e., XiJiao Union Retirement Center) were screened for eligibility through face‐to‐face interviews. Individuals were eligible for inclusion if they (1) were aged ≥ 60 years; (2) did not require special nursing care and had no history of alcoholism, smoking, irregular eating or overeating; (3) had a body mass index from 18 to 28 kg/m^2^; and (4) had been living in a long‐term care facility for more than half a year and were not expected to leave within half a year. The exclusion criteria were as follows: (1) history of stroke, severe cognitive impairment or mobility impairment; (2) severe chronic diarrhoea, nausea, vomiting, gastrointestinal obstruction or inflammatory bowel disease; (3) allergies to foods included in the intervention diets; (4) heart, liver and kidney failure; (5) hyperuricaemia or gout; (6) use of protein supplements within the 12 months prior to their enrolment in the current study or participation in other intervention programs; (7) use of antibiotics or probiotics within the past month; and (8) signs of other major medical or psychological diseases or individuals expressing the opinion that they were unsuitable for research participation.

Participants included in this subanalysis were those who provided valid faecal samples at both baseline and follow‐up (*n* = 53), with no additional selection criteria applied. To assess the representativeness of this subsample and evaluate potential selection bias, we compared their baseline demographic and clinical characteristics with those of the full study cohort (*n* = 84) and with participants who were excluded due to missing faecal samples. Comparisons were performed using independent t‐tests for continuous variables and chi‐square tests for categorical variables. No systematic differences were observed (Tables S1 and S4).

### 2.3 Intervention

The participants in the present study were randomly assigned to an intervention group or a control group. A randomization sequence was generated prior to the beginning of the study, and the sequence was stratified by gender and age (> 85 or ≤ 85 years), with block sizes of 4. The MedSci medical randomization tool was used to generate random numbers. For 12 weeks, the intervention group participants consumed three meals a day supplemented with 30 g of soy protein (10 g/meal), whereas the control group participants maintained habitual diets. Two types of soy protein were consumed by the participants, namely, textured soybean protein and soy protein powder. The soybean tissue protein was added to various Chinese dishes, whereas the soy protein powder was added to soup, congee, beverages and oatmeal. A total of 168 soy protein–rich recipes (14 per week for 12 weeks) were formulated by professional dieticians, and meal preparation was performed by chefs from the retirement centre. To assess the dietary intake of the participants, a dietitian with professional training recorded the three meals consumed by each participant 3 days a week. The SY nutrition software, which was jointly developed by the Shanghai Nutrition Quality Control Center and Fudan University School of Medicine, was used to calculate the nutritional content of the participants' diets.

### 2.4 Faecal Sample Collection

Volunteers who opted in for stool samples collection were instructed to do so at their own homes during each measurement visit at Weeks 0, 6, and 12. Collection of the stool samples was performed according to the manufacturer's instructions using the provided kits. This included a DNA/RNA Shield faecal collection tube (Zymo Research, California, USA) for microbiome studies, the OMNImet.GUT kit (ME‐200) (DNA Genotek, Ottawa, Canada) for metabolome studies. The collected samples were transported under room temperature within 24 h to the laboratory. Samples were aliquoted and stored at −80 °C when received in the laboratory.

### 2.5 DNA Isolation and Metagenomics Sequencing

Genomic DNA was extracted from samples using the ZymoBIOMICS DNA Miniprep Kit (Zymo Research, Cat# D4300) following the manufacturer's protocol. Briefly, samples were added to ZR BashingBead Lysis Tubes (0.1 and 0.5 mm) containing 750 μL of ZymoBIOMICS Lysis Solution. Mechanical lysis was performed by bead beating using optimized conditions for complete homogenization and unbiased disruption of microbial cell walls. Following bead beating, samples were centrifuged at 10 000 × g for 1 min to pellet debris.

The supernatant (up to 400 μL) was transferred to a Zymo‐Spin III‐F Filter in a Collection Tube and centrifuged at 8000 × g for 1 min. After filtration, 1200 μL of ZymoBIOMICS DNA Binding Buffer was added to the filtrate and mixed thoroughly. The mixture was then transferred to Zymo‐Spin IICR Columns for DNA binding, followed by washing steps with ZymoBIOMICS DNA Wash Buffer 1 (400 μL) and Wash Buffer 2 (700 μL and then 200 μL) to remove contaminants. DNA was eluted with 100 μL of ZymoBIOMICS DNase/RNase Free Water after a 1‐min incubation at room temperature. The eluted DNA underwent a final purification step using Zymo‐Spin III‐HRC Filters to remove PCR inhibitors.

DNA quality was assessed by 0.8% agarose gel electrophoresis to verify integrity, and concentration was determined using a Tecan F200 with Qubit dsDNA Kit (Thermofisher, Cat#Q32851). Library preparation was performed using the NEBNext Ultra II DNA Library Prep Kit for Illumina (New England BioLabs, Cat# E7645L). High‐throughput sequencing was conducted on an Illumina NovaSeq 6000 platform using the NovaSeq 6000 S4 Reagent Kit V1.5 with paired‐end 150 bp (PE150) sequencing mode.

### 2.6 Short‐Chain Fatty Acids (SCFAs) Analysis

Faecal samples were collected in ME‐200 tubes and processed according to a standardized protocol. Briefly, 450 μL of homogenized faecal sample was thawed at room temperature for 10 min and centrifuged at 15 000 g for 10 min at 4°C. The supernatant was transferred to a clean tube, and the pellet was dried in a speedvac vacuum (45°C, 30 min) and weighed for subsequent normalization. From the supernatant, 250 μL was mixed with 200 μL of ethanol, vortexed for 1 min, and centrifuged again at 15 000 g for 10 min at 4°C. The resulting supernatant was transferred to a new tube, with 45 μL aliquoted into a GC vial with insert, and acidified with 5 μL of 10% formic acid (final concentration 1% v/v).

SCFAs were analysed using gas chromatography with flame ionization detection (GC‐FID) on GC9720plus (Fuli, Taizhou) equipped with a DB‐FFAP column (30 m × 320 μm × 0.5 μm). The GC oven program started at 80°C (1 min hold), then increased at 10°C/min to 150°C, followed by 20°C/min to 240°C (10 min hold). Helium was used as carrier gas at a constant flow of 3 mL/min. Sample injection (0.5 μL) was performed in splitless mode with inlet temperature at 240°C. The FID detector operated at 240°C with hydrogen flow at 40 mL/min, air flow at 400 mL/min, and makeup flow (He) at 30 mL/min. This method allowed for detection of nine SCFAs: acetic, propionic, isobutyric, butyric, isovaleric, valeric, isocaproic, hexanoic and heptanoic acids, with retention times ranging from approximately 5.7 to 10.5 min.

### 2.7 Statistical Analysis

#### 2.7.1 Sample Size Calculation

The original sample size calculation was based on detecting a difference in lean body mass change between intervention and control groups, with a required sample size of 64 participants and a total recruitment of 84 accounting for dropout. Microbiome and SCFA analyses were performed on a subset of participants (*n* = 53) who provided faecal samples.

#### 2.7.2 Statistical Checks

Data were checked for normality visually using histograms, boxplots and quantile–quantile plots. Data were scaled before the detection of correlative longitudinal trends.

#### 2.7.3 PERMANOVA Analysis

To further evaluate the intervention effect on the microbiome composition over time, we used the ‘adonis2’ function from the vegan package (version 2.6.4) in R (version 4.3.3) to construct PERMANOVA model was applied with the group × time interaction terms as independent variables. Permutation procedure was stratified by subjects to account for longitudinal structure of the data. To test whether the means of the groups were changed in PERMANOVA, we additionally performed beta dispersion test.

#### 2.7.4 Linear Mixed Effects Model

To investigate both longitudinal microbiome and primary outcome changes, we used LMMs using the following construct.

To detect differences response between groups over time, we used fixed and random components using the following formula:

$$\text{Response}\sim \text{Group}*\text{Time}+\mathrm{Age}+\mathrm{Sex}+\mathrm{BMI}+\left(1|\text{SubjectID}\right)$$

Within each group, to detect differences across time, we used the following formula:

$$\text{Response}\sim \text{Time}+\mathrm{Age}+\mathrm{Sex}+\mathrm{BMI}+\left(1|\text{SubjectID}\right)$$

To detect correlative longitudinal trends, we used fixed and random components using the following formula:

$$\text{Response}\sim \text{variable}+\mathrm{Age}+\mathrm{Sex}+\mathrm{BMI}+\left(1|\text{SubjectID}\right)$$

Model assumptions were evaluated using a comprehensive diagnostic panel. Normality of residuals and random effects was assessed via Q–Q plots. Homoscedasticity and linearity were examined using residual‐versus‐fitted plots.

For correlation trends involving multiple species and pathways, the Benjamini–Hochberg (BH) method was used to calculate *q* values for the variable coefficients, with a cutoff of 0.05.

## 3 Results

### 3.1 Baseline Characteristics of Participants and Macronutrients Intake Analysis

Table 1 shows the baseline demographic and clinical characteristics of the 53 participants who provided faecal samples for this subanalysis. The mean age was 84.67 years, with 62.3% female participants, and the mean BMI was 23.38 kg/m^2^. No significant differences were observed between the two groups for age, sex distribution, height, weight, handgrip strength, SPPB scores or blood biomarkers (including IL‐6, IGF‐1, Hs‐CRP and 25‐OH‐VD). Participants in the intervention group had significantly higher BMI (24.57 vs. 22.35 kg/m^2^, *p* = 0.011), larger calf circumference (CC) (34.41 vs. 32.48 cm, *p* = 0.023) and faster performance in the five‐time chair stand test (14.73 vs. 18.03 s, *p* = 0.038).

**TABLE 1:** Baseline measurements for intervention group (IG) and control group (CG).

Clinical characteristics	Intervention (*n* = 25)	Control (*n* = 28)	*p*
**General information** Mean (SD)			
Sex F (%)	16 (64.0)	17 (60.7)	1
Age	83.76 (6.39)	85.50 (7.09)	0.355
**Anthropometric parameters** Mean (SD)			
Height (m)	1.56 (0.07)	1.58 (0.09)	0.396
Weight (kg)	59.86 (7.68)	55.90 (11.59)	0.155
BMI (kg/m^2^)	24.57 (2.23)	22.35 (3.64)	**0.011**
**Muscle function index** Mean (SD)			
CC (cm)	34.41 (1.83)	32.48 (3.72)	**0.023**
SMI (kg/m^2^)	6.26 (0.73)	5.97 (1.15)	0.283
HGS (kg)	19.50 (4.80)	18.73 (5.95)	0.606
6‐m walk (s)	10.72 (5.95)	10.74 (5.03)	0.993
5‐time chair stand (s)	14.73 (3.83)	18.03 (6.85)	**0.038**
SPPB	7.92 (2.75)	7.21 (2.83)	0.363
**Blood biomarkers** Mean (SD)			
IL‐6 (pg/mL)	7.87 (10.15)	6.58 (7.39)	0.596
IGF‐1 (ng/mL)	97.32 (29.90)	85.25 (25.87)	0.121
Hs‐CRP (mg/L)	1.35 (1.38)	2.19 (2.62)	0.155
25‐OH‐VD (μg/L)	17.44 (8.12)	16.77 (5.63)	0.728

Abbreviations: 25‐OH‐VD, 25‐hydroxyvitamin D; CC, calf circumference; HGS, handgrip strength; Hs‐CRP, high‐sensitivity C‐reactive protein; IGF‐1, insulin‐like growth factor 1; IL‐6, interleukin‐6; SMI, skeletal muscle index; SPPB, short physical performance battery.

To examine whether this subsample was representative of the overall study cohort, we compared their characteristics with those of the full cohort (Table S1). No significant differences were observed across age, sex distribution, BMI or functional status, indicating that the faecal sample subgroup was broadly comparable to the full study population. Analysis of dietary intake showed no significant difference in total energy consumption between the control and intervention groups over the 12‐week period (*p*_(time×group)_ = 0.23; Table S2). While total protein intake increased significantly in the intervention group (*p* = 0.01), this change was primarily attributable to the substantial increase in soy protein intake (*p* < 0.001).

### 3.2 Changes in Muscle Function and Inflammatory Factors

**FIGURE 1:**
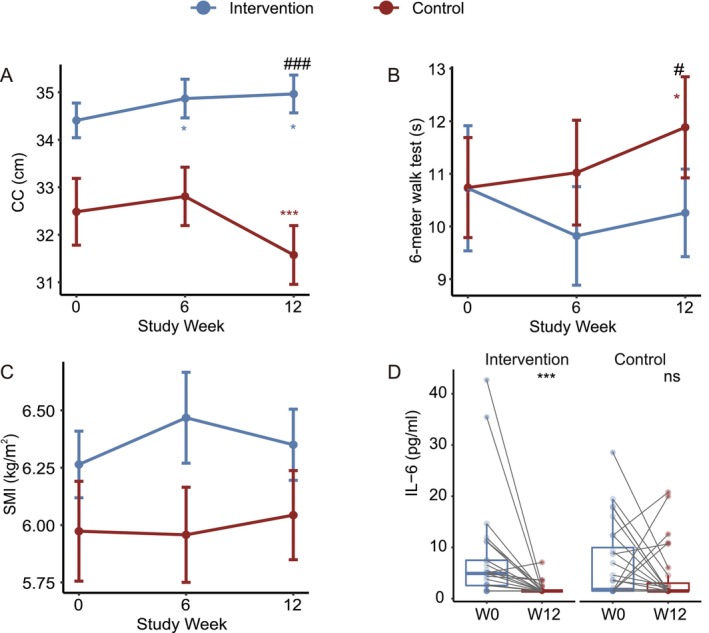
Changes in muscle health outcomes and blood biomarkers. (A–C) Changes from baseline in (A) CC, (B) 6‐m walk time, and (C) SMI. Data presented as mean ± standard error. ^#^ *p*_(time×group)_ < 0.05, denote *p* values for the time × group interaction coefficient in the LMM model (the change in 6‐m walk time approached statistical significance, *p*_(time×group)_ = 0.05); *p_(time)_ < 0.05, **p_(time)_ < 0.01 denote *p* values for the time coefficient within the same group. (D) Paired boxplot shows the changes in IL‐6 for each subject. The line inside each box is the median, and the box edges show the range where the middle 50% of data lies. Whiskers extend to the smallest and largest values within 1.5 times the interquartile range. Outliers are shown as individual points. Asterisks indicate significant differences based on paired Wilcoxon signed‐rank tests. Abbreviations: CC, calf circumference; SMI, skeletal muscle index; IL‐6, interleukin‐6.

After 12 weeks of intervention, CC showed a decreasing trend in the control group, while it was maintained in the intervention group, with a statistically significant difference between the two groups (*p*_(time×group)_ < 0.001; Figure 1A). Changes in 6‐m walk time diverged between groups, with a trend toward reduction in the intervention group and increase in the control group from baseline, approaching statistical significance (*p*_(time×group)_ = 0.05; Figure 1B). Skeletal muscle index (SMI) demonstrated an upward trend in the intervention group and a downward trend in the control group, although the between‐group difference was not significant (Figure 1C). Both groups exhibited similar temporal patterns in Short Physical Performance Battery (SPPB), handgrip strength (HGS) and five‐time chair stand test performance across the study period (Figure S1A–C). Analysis of inflammatory markers revealed differential responses between groups. Interleukin‐6 (IL‐6) levels showed a significant decrease in the intervention group (paired Wilcoxon test, *p* < 0.001) while remaining unchanged in the control group (Figure 1D). Both groups exhibited significant reductions in 25‐OH Vitamin D levels (Figure S1D). Neither hypersensitive C‐reactive protein (hs‐CRP) nor insulin‐like growth factor 1 (IGF‐1) showed significant changes in either group throughout the study period (Figure S1E,F).

Additional sex‐stratified analyses (Table S3) demonstrated similar intervention trends in both males and females.

### 3.3 Changes in Gut Microbiota

**FIGURE 2:**
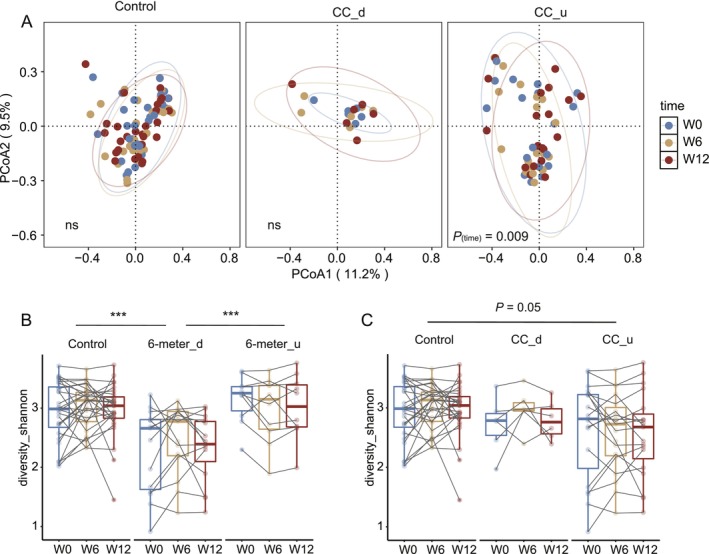
Changes in overall structure of gut microbiome. (A) Principal coordinate analysis of Bray–Curtis distances. The axes are labelled with the per cent variance explained. (B,C) Paired boxplot shows the changes in Shannon index for each subject. Asterisks indicate significant differences between groups or sub groups. ***p < 0.001 denote *p* values for the group coefficient. ‘CC_d’ and ‘CC_u’ indicate participants whose calf circumference decreased/remained stable or increased, respectively; ‘6‐m_d’ and ‘6‐m_u’ indicate participants whose 6‐m walking time decreased or remained stable/increased, respectively, after the intervention.

There were no significant changes over time in the overall gut microbiota structure in both the control group and the intervention group (Figure S2A). However, among the subjects in the intervention group who experienced an increase in CC, the gut microbiota showed significant changes over time (*p*_(time)_ = 0.009; Figure 2A). The Shannon index also did not show significant changes over time in either group (Figure S2B). However, compared to the subjects in the intervention group whose 6‐m walk time remained the same or increased and the control group, the subjects in the intervention group with decreased 6‐m walk time had a significantly lower Shannon index (*p* < 0.001; Figure 2B). Among the subjects with increased CC, the Shannon index was also nearly significantly lower (*p* = 0.05; Figure 2C).

Several species showed significant changes over time only in the intervention group or in the control group.

At the genus level, we identified three genera that showed significantly different changes over time between the two groups: *Ruminococcaceae unclassified* (*p*_(time*group)_ = 0.03), *Monoglobus* (*p*_(time*group)_ = 0.005) and *Alistipes* (*p*_(time*group)_ = 0.01) (Figure S3A–C).

**FIGURE 3:**
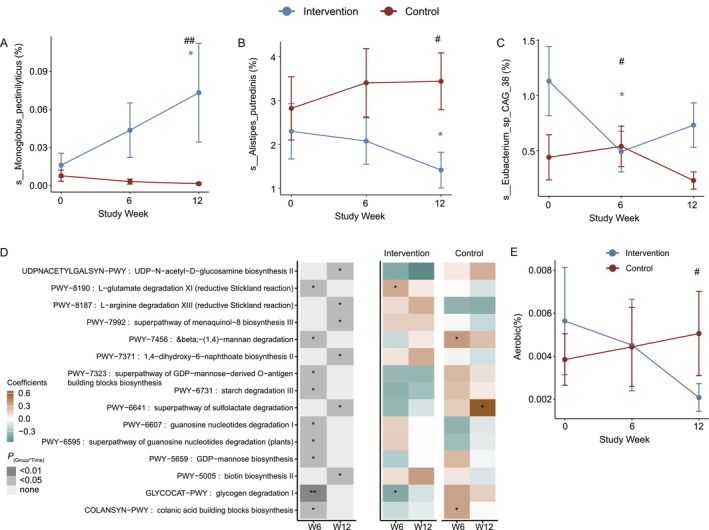
Changes in gut microbiota species and functions. (A–C) Lineplots show the temporal changes in specific species. Data presented as mean ± standard error. ^#^ *p*_(time×group)_ < 0.05, ^##^ *p*_(time×group)_ < 0.01 denote *p* values for the time × group interaction coefficient in the LMM model.*p_(time)_ < 0.05 denote *p* adjusted values for the time coefficient within the same group. Asterisks denote raw *p* values without multiple‐comparison correction; results did not pass BH‐FDR across pathways (*q* ≥ 0.05). D: Heatmap of *p* values and coefficients of function pathway alterations across the whole study period. Left panel: *p* values for the time × group interaction coefficient in the LMM model; *p_(time×group)_ < 0.05; **p_(time×group)_ < 0.01. Right panel: *p* values and coefficients for the time term in the LMM model performed separately for each group; *p_(time)_ < 0.05. Asterisks denote raw *p* values without multiple‐comparison correction; results did not pass BH‐FDR across pathways (*q* ≥ 0.05). (E) Temporal changes in aerobic bacteria abundance in two groups across the 12‐week study period. Data represented as mean ± standard error. #*p*_(time×group)_ < 0.05 denote *p* values for the time × group interaction coefficient in the LMM model. Significance is based on raw *p* values.

At the species level, 25 species showed significant changes over time between the two groups (*p*_(time*group)_ < 0.05). Among them, *Monoglobus pectinilyticus* , the first human gut bacterium found to specialize in degrading and utilizing pectin, showed a significant increase over time in the intervention group, consistent with the trend observed at the genus level (Figure 3A). Similarly, *Alistipes putredinis* , belonging to the genus *Alistipes*, and *Eubacterium_sp_CAG_38* decreased significantly over time in the intervention group (Figure 3B,C).

We also found that one branched‐chain amino acid degrading species, *Parabacteroides merdae* , showed a significant increase in the control group, while it decreased marginally significantly in the intervention group (Figure S3D). Notably, the abundance of *Parabacteroides merdae* was significantly decreased in subjects whose CC and SMI index increased in the intervention group, compared to subjects whose CC and SMI index were unchanged or decreased (Figure S4A,B). *Eubacterium_sp_CAG_38* and *Dorea formicigenerans* showed a similar change trend (Figure S4C,D).

On the other hand, we also observed an enrichment of some short‐chain fatty acid‐producing bacteria in the gut in the intervention group (*p*_(time)_ < 0.05), such as *Agathobaculum butyriciproducens* and *Roseburia faecis* (Figure S3E,F).

### 3.4 Functional Alterations in the Gut Microbiome

The intervention also significantly altered specific functional pathways in the gut microbiota compared to the control group (Figure 3D). We observed enrichment of pathways involved in the anaerobic degradation of glutamate (*p*_(time×group)_ < 0.05) and arginine (*p*_(time×group)_ < 0.05, *p*_(time)_ < 0.05). The enhancement of these anaerobic metabolic pathways indicates an expansion of anaerobic bacterial populations in the gut microbiota. Notably, the glutamate degradation pathway plays a role in short‐chain fatty acid production. Additionally, pathways related to vitamin biosynthesis, including menaquinones and biotin, showed significant enrichment (*p*_(time×group)_ < 0.05).

Conversely, several carbohydrate metabolism pathways were significantly depleted in the intervention group's gut microbiome, including β‐(1,4)‐mannan, starch (*p*_(time×group)_ < 0.05) and glycogen degradation (*p*_(time×group)_ < 0.05, *p*_(time)_ < 0.05). The intervention also led to reduced activity in inflammatory pathways, specifically those related to lipopolysaccharide (LPS) biosynthesis, including colanic acid building blocks biosynthesis, GDP‐mannose biosynthesis, UDP‐*N*‐acetyl‐D‐glucosamine biosynthesis and the superpathway of GDP‐mannose‐derived O‐antigen building blocks biosynthesis (*p*_(time×group)_ < 0.05) (Figure 3D). Furthermore, phenotype prediction revealed a notable decline in aerobic bacteria in the intervention group over the 12‐week study period, while the control group showed a slight increase, suggesting a shift in the metabolic capacity of the gut microbiota (Figure 3E).

### 3.5 Changes in Intestinal SCFAs

**FIGURE 4:**
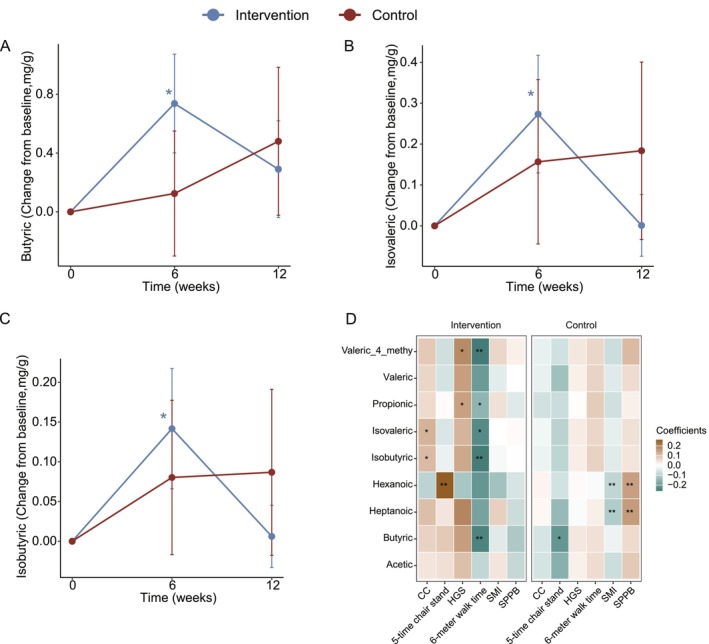
Changes in gut microbiota species and functions. (A–C) Lineplots show the temporal changes in butyric acid; isobutyric acid and isovaleric acid. Data represented as mean ± standard error. *p_(time)_ < 0.05 denote raw *p* values for the time coefficient within the same group. (D) Heat map showing the associations of SCFAs with the muscle health outcomes; *q < 0.05; **q < 0.01. Significance based on Benjamini–Hochberg FDR correction across correlations within each group, with a cutoff of 0.05. Abbreviations: CC, calf circumference; HGS, handgrip strength; SMI, skeletal muscle index; SPPB, short physical performance battery.

Although the group × time interaction was not statistically significant (butyric acid = +0.57 [95% CI: −0.53, 1.68], *p* = 0.31; isobutyric acid = +0.05 [−0.18, 0.28], *p* = 0.70; isovaleric acid = +0.08 [−0.38, 0.55], *p* = 0.73), post hoc analyses showed that within the intervention group, concentrations of butyric acid, isobutyric acid and isovaleric acid increased significantly at Week 6 compared with baseline (butyric acid = +0.72 [95% CI: 0.07, 1.37], *p* = 0.03; isobutyric acid = +0.13 [0.02, 0.25], *p* = 0.03; isovaleric acid = +0.26 [0.03, 0.49], *p* = 0.03), followed by a partial decline toward baseline by Week 12, whereas within the control group, no significant temporal changes were observed (butyric acid = +0.15 [95% CI: −0.71, 1.01], *p* = 0.73; isobutyric acid = +0.09 [−1.02, 0.28], *p* = 0.36; isovaleric acid = +0.18 [−0.21, 0.58], *p* = 0.37; Figure 4A–C). Other six fatty acids had no significant change in both groups (Figure S5). Associations between SCFAs and muscle function were also analysed. We observed that throughout the entire intervention period, there were more associations in the intervention group compared to the control group. Specifically, in addition to butyric acid, three BCFAs (4‐methyl valeric acid, isobutyric acid and isovaleric acid) were negatively associated with 6‐m walk time, while isobutyric acid and isovaleric acid were positively associated with CC. These correlations were not observed in the control group (Figure 4D).

### 3.6 Associations Between Gut Microbiota Features and Muscle Function Parameters

**FIGURE 5:**
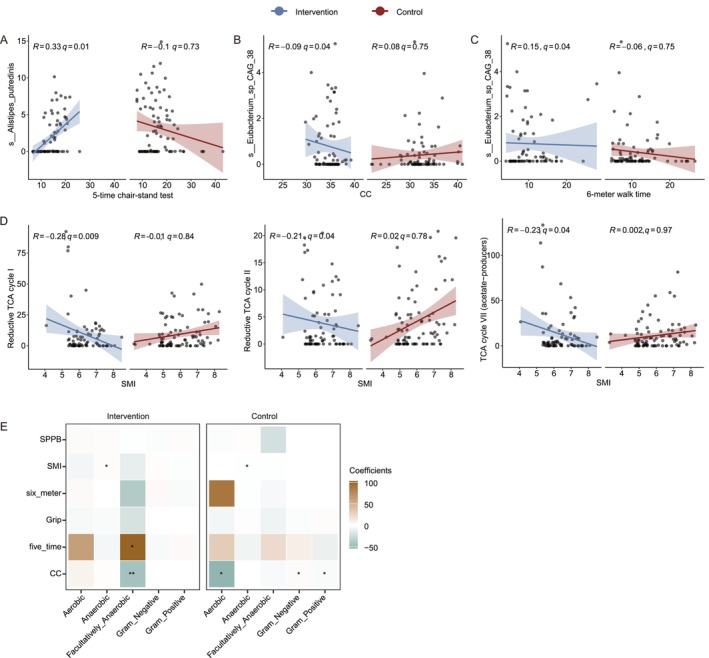
Associations between gut microbiota features and muscle function parameters. (A–D) Scatter plots displaying correlations between bacterial species abundance or metabolic pathway activity and muscle function measurements in intervention (blue) and control (red) groups. (A) *Alistipes putredinis* abundance positively correlates with five‐time chair‐stand test in the intervention group (*R* = 0.33, *q* = 0.01) but not in control group. (B) Eubacterium_sp_CAG_38 shows negative association with calf circumference (CC) in the intervention group (*R* = −0.09, *q* = 0.04). (C) Eubacterium_sp_CAG_38 positively correlates with 6‐m walk time in the intervention group (*R* = 0.15, *q* = 0.04). (D) TCA cycle pathways (Reductive TCA I, Reductive TCA II, and TCA VII) show significant negative correlations with skeletal muscle index (SMI) in the intervention group. Significance based on Benjamini–Hochberg FDR correction across correlations within each group, with a cutoff of 0.05. The smooth curve represents the trend line fitted using linear model, and the shaded area indicates the 95% confidence interval. The *R* values and *q* values displayed in the figure were calculated based on linear mixed effects models (LMM) performed separately within each group. (E) Heatmap showing correlation coefficients between different bacterial phenotypes and muscle function parameters in both intervention and control groups. Significance is based on raw *p* values (*p < 0.05; ** *p* < 0.01). Abbreviations: CC, calf circumference; HGS, handgrip strength; SMI, skeletal muscle index; SPPB, short physical performance battery.

We then investigated the association between muscle function and gut microbiota species and functional pathways. *Alistipes putredinis* , which was found to be reduced in the intervention group, is positively correlated with the five‐time chair‐stand test time in the intervention group, but not in the control group (Figure 5A, Figure S6A). Another species found to be reduced by the intervention, *Eubacterium_sp_CAG_38*, was negatively associated with CC in the intervention group (Figure 5B, Figure S6A), while it was positively associated with 6‐m walk time (Figure 5C, Figure S6A).

In terms of the functional pathways, there is a more complex connection between pathways and muscle functions in the intervention group (Figure S3B). Notably, we found that all pathways involved in the TCA cycle were negatively associated with SMI (Figure 5D, Figure S6B). Furthermore, both sulfur‐containing amino acid biosynthesis pathways (cysteine and methionine) showed negative correlations with SMI in the intervention group (Figure S6C,D). The lysine degradation pathway negatively correlated with CC, while lysine biosynthesis was positively associated with SMI (Figure S6E,F). The glycogen degradation pathway was also negatively associated with SMI in the intervention group (Figure S6G), whereas facultative anaerobic bacteria demonstrated a strong positive correlation with the time of the five‐time chair stand test and showed a negative correlation with the CC (Figure 5L).

## 4 Discussion

In this study, we investigated whether a soy protein–rich meal intervention would help older adults in a long‐term care facility to maintain or improve their muscle health. The primary outcome of this trial has already been published [25]. In this article, we focused on whether the gut microbiota could be linked to the improvements in muscle health induced by the soy protein–rich diet.

A substantial body of prior research has elucidated the alterations in the composition and functionalities of the gut microbiota in individuals with sarcopenia. These findings substantiate the hypothesis that there is a correlation between the gut microbiome and muscle health. One study observed an overall reduction in microbial diversity in sarcopenia and possible sarcopenia samples. The genera *Lachnospira*, *Fusicantenibacter*, *Roseburia*, *Eubacterium* and *Lachnoclostridium*—known butyrate producers—were significantly less abundant in sarcopenia and possible sarcopenia subjects while Lactobacillus was more abundant [26]. In addition, a study of fluctuated temperature accelerates muscle atrophy and dampens exercise performance in mid‐aged male mice showed that fluctuated temperature alters the microbiota composition with increased levels of *Parabacteroides distasonis* , *Duncaniella dubosii* , and decreased levels of *Candidatus Amulumruptor* , *Roseburia* , *Eubacterium* and *Eubacterium* supplementation alleviates muscle atrophy and dysfunction induced by fluctuated temperature [27].

In our study, even though there were no significant changes in the overall gut microbiota structures in either the intervention or control groups over time, we found that there was a significant change in individuals with increased CC in the intervention group. The Shannon index demonstrated minimal fluctuation over time; however, it was observed to be relatively low in individuals with reduced 6‐m walk time and increased CC. A study comparing the effects of low glycinin soymilk (LGS), conventional soymilk (S) and bovine milk (M) on the gut microbiome of overweight and obese men found that, after a 3‐month intervention, gut microbiota diversity decreased in all three groups [28]. One possible reason why greater muscle health improves in individuals with low diversity is that low diversity makes the gut microbiota more susceptible to dietary interventions thereby producing changes that are favourable to muscle health.

At individual species level, it was observed that the intervention group exhibited a notable increase in the relative abundance of *Roseburia faecis* and *Agathobaculum butyriciproducens* , two species known to produce SCFAs [29, 30]. We also found *Monoglobus pectinilyticus* , a pectin‐degrading specialist bacterium in the human colon [31], significant increase in the intervention group. Previous studies [26, 27, 32] have shown consistent results, supporting the idea that our dietary intervention was effective in reversing the alterations in sarcopenia gut microbiota and augmenting the prevalence of some beneficial bacteria. Conversely, the intervention group demonstrated a significant decline in *Parabacteroides merdae* , particularly among individuals with elevated CC and SMI, whereas the control group exhibited a notable increase in this microbe. It has been demonstrated that the elevated abundance of this microbe, caused by a Ganoderma meroterpene derivative (GMD), enhances the breakdown of intestinal BCAAs, thereby ameliorating atherosclerosis [33]. We also observed a significant reduction in the *Eubacterium_sp_CAG_38*, *Alistipes putredinis* and genus *Ruminococcaceae unclassified* in the intervention groups. It has been demonstrated that *Alistipes putredinis* contributes to weight loss through exercise and is associated with autism [34, 35]. This is partly consistent with previous findings showing that soy protein increases *Ruminococcus*, *Lactobacilli* and *Bacteroidetes* in the gut of animal models [36, 37]. Further studies in human subjects are needed to improve the consistency of these findings.

Furthermore, our findings revealed that specific functions of the gut microbiome exhibited notable alterations. Firstly, the glutamate degradation pathway, which is the acetate and butyrate production pathway [38], was increased in the intervention group. Given that glutamate is the most abundant amino acid in our soy protein and that the butyrate‐producing species were raised after the intervention, this is a reasonable and consistent result. We also found that vitamin biosynthesis pathways (menaquinones and biotin) were enriched in the intervention group. Vitamins are frequently identified as being crucial to human health. Biotin (vitamin B7) is an essential cofactor for carboxylase enzymes involved in carbohydrate, fat and protein metabolism, thereby contributing to ATP production required for muscle contraction and recovery. In addition, it supports protein synthesis and muscle fibre repair following exercise or muscle breakdown. Previous studies have also suggested that adequate biotin availability may help alleviate muscle pain or cramps and improve muscle function [39], although the evidence remains limited, providing one possible mechanistic link between gut microbiota alterations and muscle health. However, their relationship to skeletal muscle health remains an area that requires further investigation.

Numerous studies have identified functional alterations in the gut microbiota of individuals with sarcopenia. A significant elevation in metabolic pathways, including the TCA cycle, LPS biosynthesis, protein folding and related processing, was observed in the sarcopenia population [26, 32]. However, our findings indicate a significant reduction in both the LPS synthesis pathway and the TCA cycle pathway in the intervention group, which suggests a reduction in the number of aerobic and pathogenic bacteria present in the gut. This result is consistent with a significant decrease in IL‐6 in the intervention group. The reduction in microbial TCA cycle activity may reflect a metabolic shift toward increased short‐chain fatty acid (SCFA) production, which has been shown to provide additional energy substrates for skeletal muscle and modulate muscle metabolism. Moreover, the observed decrease in LPS biosynthesis pathways may indicate a reduced pro‐inflammatory potential of the microbiota, which could help mitigate chronic low‐grade inflammation, a known contributor to muscle catabolism in older adults [40]. These findings indicate that our soy protein–rich dietary intervention may have the potential to reduce the presence of pathogenic bacteria, attenuate inflammatory responses and enhance gut and muscle health. Although causality cannot be established within the scope of our metagenomic analysis, these findings suggest plausible mechanisms through which the gut microbiome may interact with host muscle physiology and highlight potential targets for future mechanistic studies.

The correlation between altered intestinal microbiota species and function with muscle function may indicate a role for intestinal microbiota in muscle function improvement. We observed that two reduced species induced by intervention were positively associated with five‐time chair‐stand test time and 6‐m walk time, while one of them was negatively associated with CC. This suggests that a decline in these two species may lead to an improvement in muscle mass and function. The negative association between lysine degradation pathway and CC and the positive association between lysine biosynthesis pathway and SMI are consistent with previous study results that showed that lysine‐degrading metabolite aminoadipic acid mediates FT‐induced muscle atrophy and dysfunction [26]. Notably, all pathways involved in TCA cycle were negatively associated with SMI. This suggests that the reduction of aerobic bacteria may have a contributory role in the observed increase in SMI.

Based on these alterations in the gut microbiota, we also saw a corroborated increase in levels of butyric acid and two branched‐chain fatty acids in the intervention group. A number of studies have revealed that individuals with sarcopenia exhibit reduced levels of butyric acid in the intestine [20, 41]. The correlation between butyric acid and branched‐chain fatty acids with muscle function found in our study also demonstrates the role of short‐chain fatty acids in maintaining skeletal muscle health, which has been extensively demonstrated in previous research [21, 42].

While our study demonstrates a potential link between the gut microbiome and muscle health, the present study has several limitations. This study employed an open‐label design as it was not feasible to blind the participants or the outcome assessors because the appearance and taste of the intervention and control meals differed noticeably, making a placebo control impractical. Consequently, performance and reporting biases cannot be entirely ruled out and must be considered when interpreting the findings. We acknowledge the potential for selection bias given that only a subset of participants provided faecal samples for analysis. However, comparative analyses indicated no significant differences in baseline demographic characteristics between participants included in the faecal sample analysis and those excluded (Table S4). Although baseline 6‐m walk test differed between the two groups, the direction and magnitude of change were highly consistent with those observed in the overall study population (*n* = 84), suggesting that this difference is unlikely to have materially affected the interpretation of our findings. Our study did not consider negative control samples in the metagenomic workflow but had included a positive control (ZymoBIOMICS Microbial Community Standard) to ensure sequencing accuracy. The microbiome analysis included only 53 participants who provided faecal samples, which may reduce statistical power and increase the risk of Type II errors. Future larger studies are needed to confirm these findings. The participants in this study included relatively healthy, nonfrail older adults residing in a single long‐term care facility in Shanghai. As such, caution should be exercised when generalizing these findings to frailer older adults, community‐dwelling populations or those from different cultural or dietary contexts. Differences in health status, living environment and habitual diet may influence the intervention's effectiveness and gut microbiome response. Future studies including more diverse and representative populations are needed to confirm the broader applicability of our results.

In conclusion, our results suggest that our soy protein–rich dietary intervention can lead to improvements in various aspects of gut microbiota, which play a crucial role in enhancing muscle function. This highlights the role and value of soy protein in preventing and improving muscle health in older adults. Furthermore, these results demonstrate the impact of soy protein intervention on gut microbiota, offering novel insights into the relationship between gut microbiota and sarcopenia. A comprehensive understanding of the interplay between the gut microbiota and skeletal muscle health can pave the way for the development of more effective and targeted strategies for the prevention and treatment of sarcopenia.

## Author Contributions

J.S. and Y.J. designed the study. X.W. and K.J.L. analysed the data. Y.M. and L.Z. conducted experiments. Y.C. designed the meals. J.S., Y.C. and J.G. enrolled volunteers and conducted the clinical trial. X.W. and K.J.L. wrote the manuscript. All authors significantly contributed to interpreting the results, critically revised the manuscript for important intellectual content and approved the final manuscript.

## Conflicts of Interest

The authors declare no conflicts of interest.

## Supporting information


**Data S1:** Supplementary Information.

**Table S1:** Baseline characteristics of participants included in the faecal sample analysis the full study cohort.
**Table S2:** Total energy and macronutrient intake of the 2 groups. Data is presented as mean +/− SEM.
**Table S3:** Outcomes stratified by sex. Data is presented as mean +/− SEM.
**Table S4:** Baseline characteristics of participants included vs. not included from the faecal sample analysis.

**Figure S1:** Changes in muscle health outcomes and blood biomarkers A‐C: Changes from baseline in (A) SPPB, (B)HGS, (C) 5‐time chair stand test. Data presented as mean ± standard error. *p (Time) < 0.05, **p (Time) < 0.01 denote p‐adjusted values for the time coefficient within the same group. D‐F: Paired boxplot shows the changes in (D) 25‐OH‐VD, (E) hsCRP and (F) IGF‐1 for each subject. The line inside each box is the median, and the box edges show the range where the middle 50% of data lies. Whiskers extend to the smallest and largest values within 1.5 times the interquartile range. Outliers are shown as individual points. Asterisks indicate significant differences.
**Figure S2:** Changes in overall structure of gut microbiome A: Principal coordinate analysis of Bray‐Curtis distances. The axes are labelled with the percent variance explained. B: Paired boxplot shows the changes in Shannon index over time for each subject in both groups.
**Figure S3:** Changes in gut microbiota genus and species Lineplots show the temporal changes in specific genus and species. Data presented as mean ± < 0.01 denote p values for the time×group interaction coefficient in the LMM model.*p (Time) < 0.05, **p (Time) < 0.01 denote p values for the time coefficient within the same group.
**Figure S4:** Changes in specefic species < 0.05, ##*p* (Time×group) < 0.01 denote p values for the time×group interaction coefficient in the LMM model.*p (Time) < 0.05, **p (Time) < 0.01 denote p values for the time coefficient within the same group. “CC_d” and “CC_u” indicate participants whose calf circumference decreased/remained stab le or increased, respectively; “SMI_d” and “SMI_u” indicate participants whose SMI decreased/rema ined stable or increased, respectively.
**Figure S5:** Changes in faecal SCFAs A‐F: Paired boxplot shows the changes in six SCFAs for each subject. The line inside each box is the median, and the box edges show the range where the middle 50% of data lies. Whiskers extend to the smallest and largest values within 1.5 times the interquartile range. Outliers are shown as individual points.
**Figure S6:** Associations between gut microbiota and muscle health outcomes A‐B: Heatmap of p values and coefficients of associations between species (A) or payhways (B) and muscle health outcomes across the whole study period. Only correlations with p‐values < 0.05 for time*group terms in the LMM model are shown. *q < 0.05; **q < 0.01; ***q < 0.001 gives the p‐adjusted values for the variable association coefficient in the LMM model performed separately in two groups. C‐G: Scatter plots displaying correlations between metabolic pathway activity and muscle function measurements in intervention (blue) and control (red) groups. (C‐D) Sulfur‐containing amino acid biosynthesis pathways (L‐cysteine and L‐methionine) negatively correlate with SMI in the intervention group. (E) Glycogen degradation pathway negatively correlates with SMI in the intervention group (R = ‐0.24, q = 0.03). (F) L‐lysine biosynthesis positively associates with SMI in the intervention group (R = 0.28, q = 0.03). (G) L‐lysine degradation pathway negatively correlates with CC in the intervention group (R = ‐0.15, q = 0.03). The smooth curve represents the trend line fitted using Linear Model, and the shaded area indicates the 95% Confidence Interval. The R values and q values displayed in the figure were calculated based on Linear Mixed effects Models (LMM) performed separately within each group.
