# Supplementary figures and images for: The Effects of Soy Protein–Rich Meals on Muscle Health of Older Adults Are Linked to Gut Microbiome Modifications
Source: J Cachexia Sarcopenia Muscle. 2026 Jan 25;17(1):e70212. doi: 10.1002/jcsm.70212 (PMC12833501; doi:10.1002/jcsm.70212)

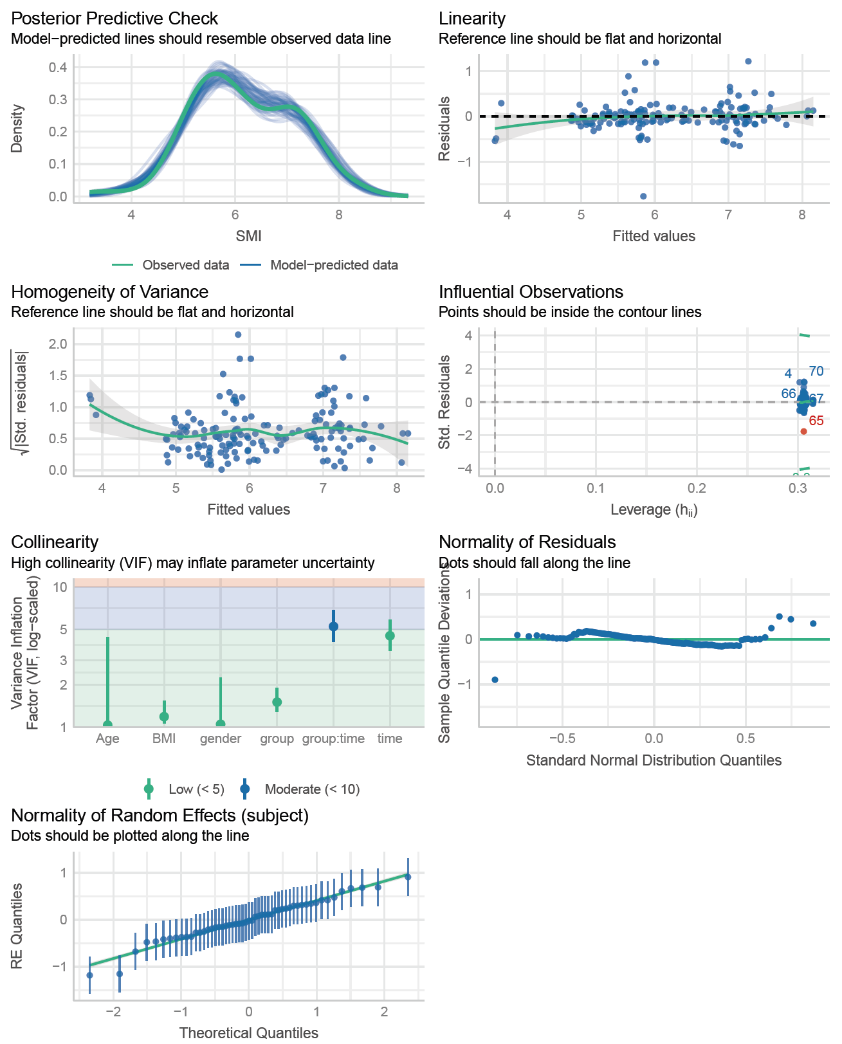

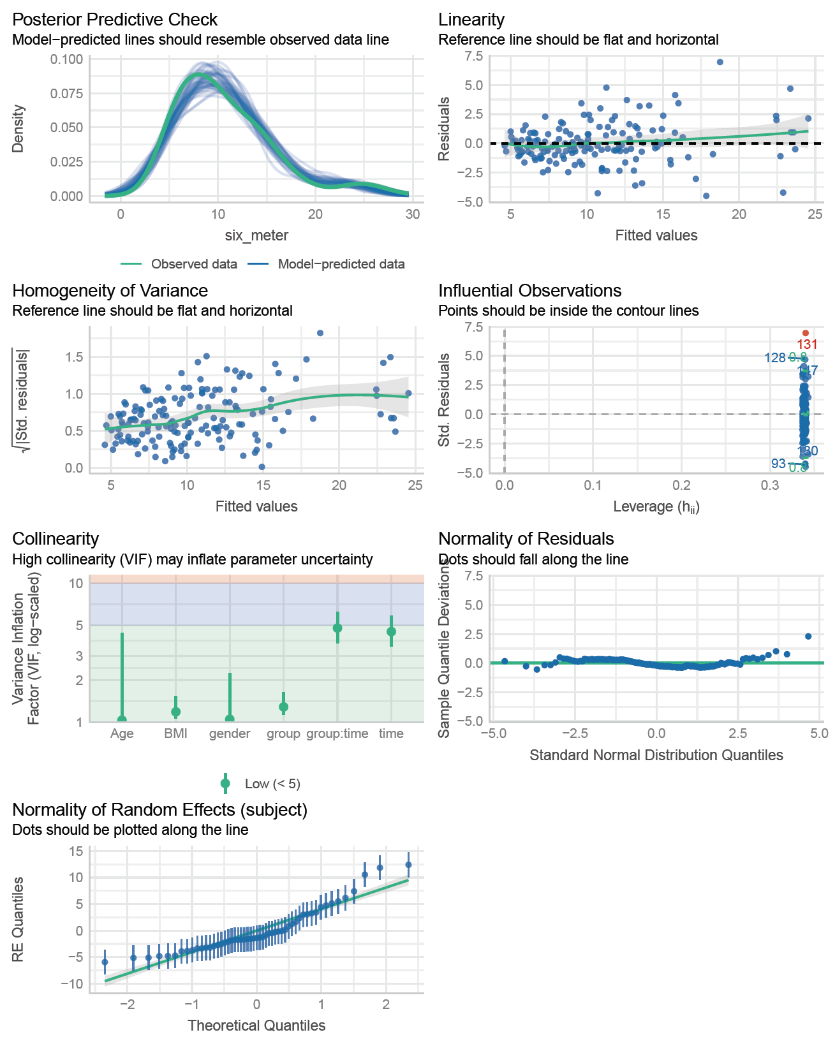

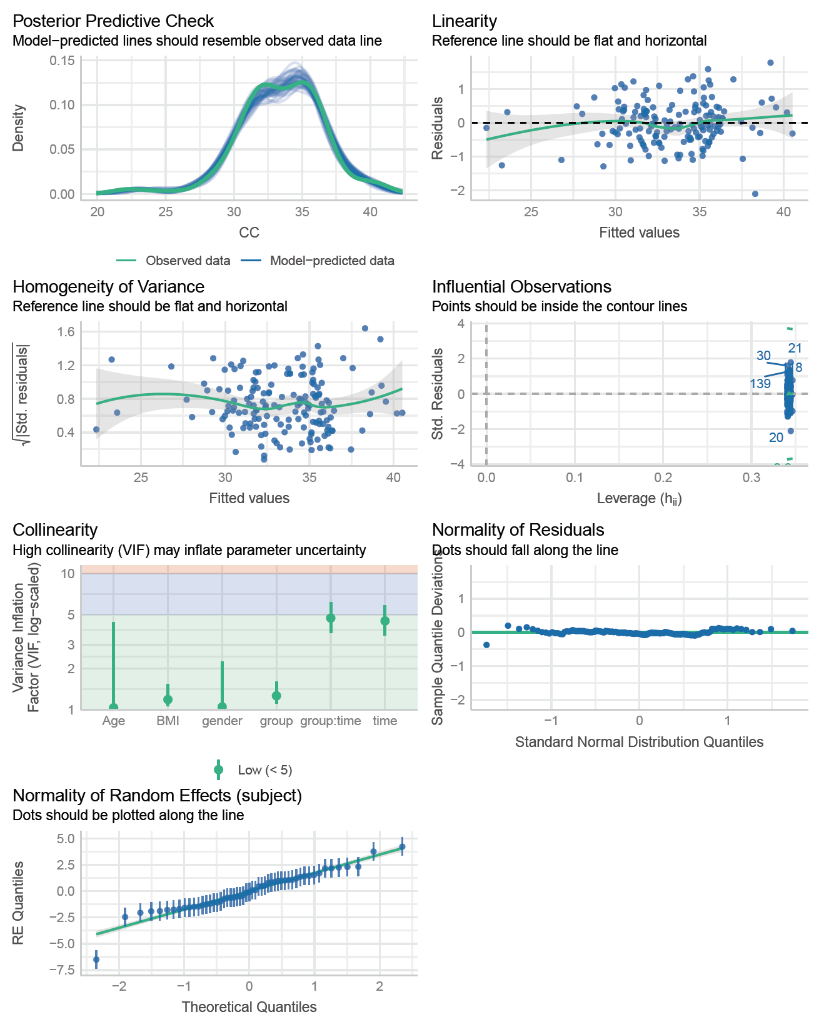


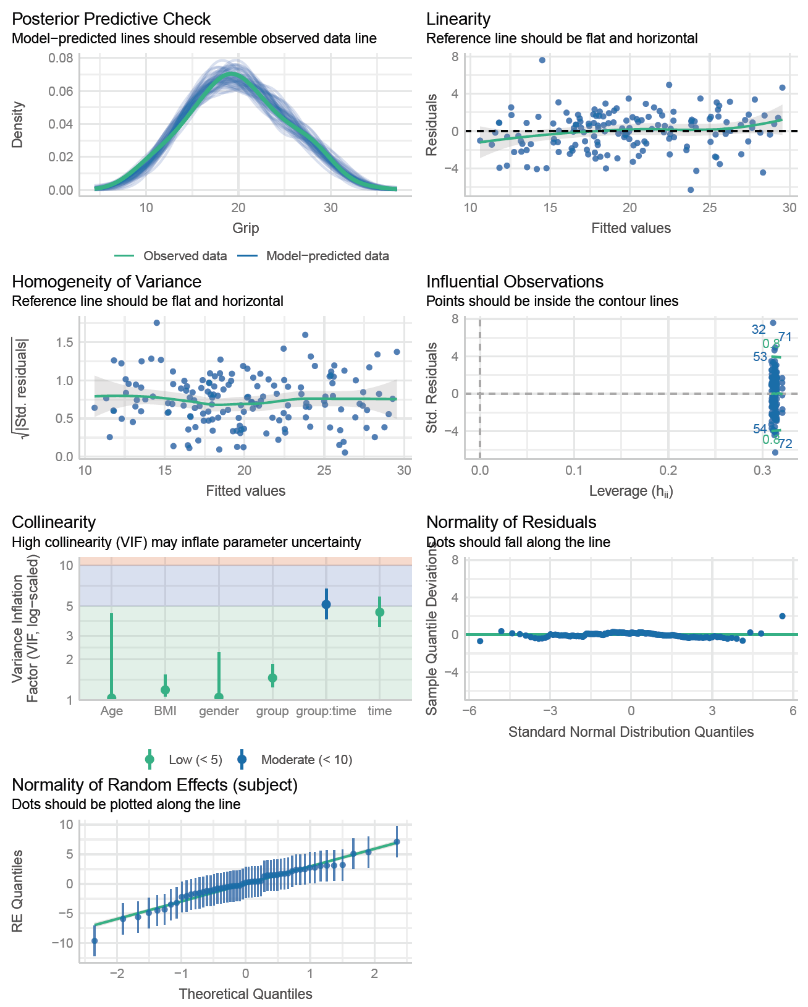


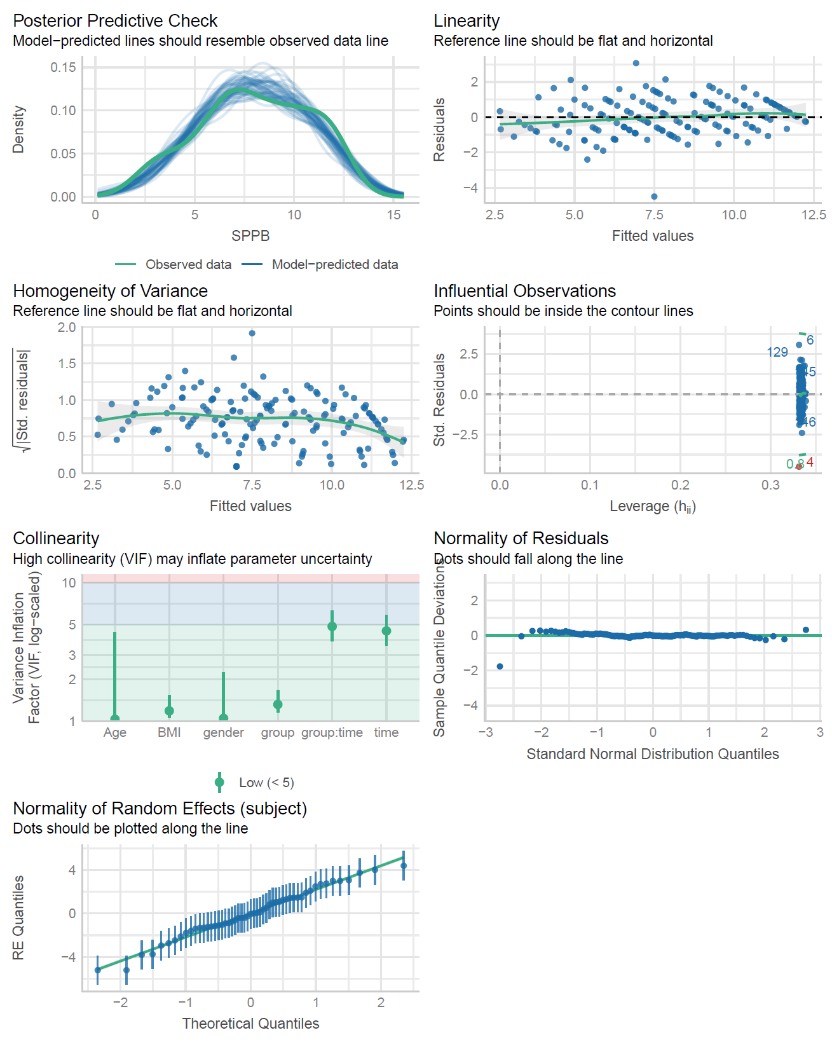

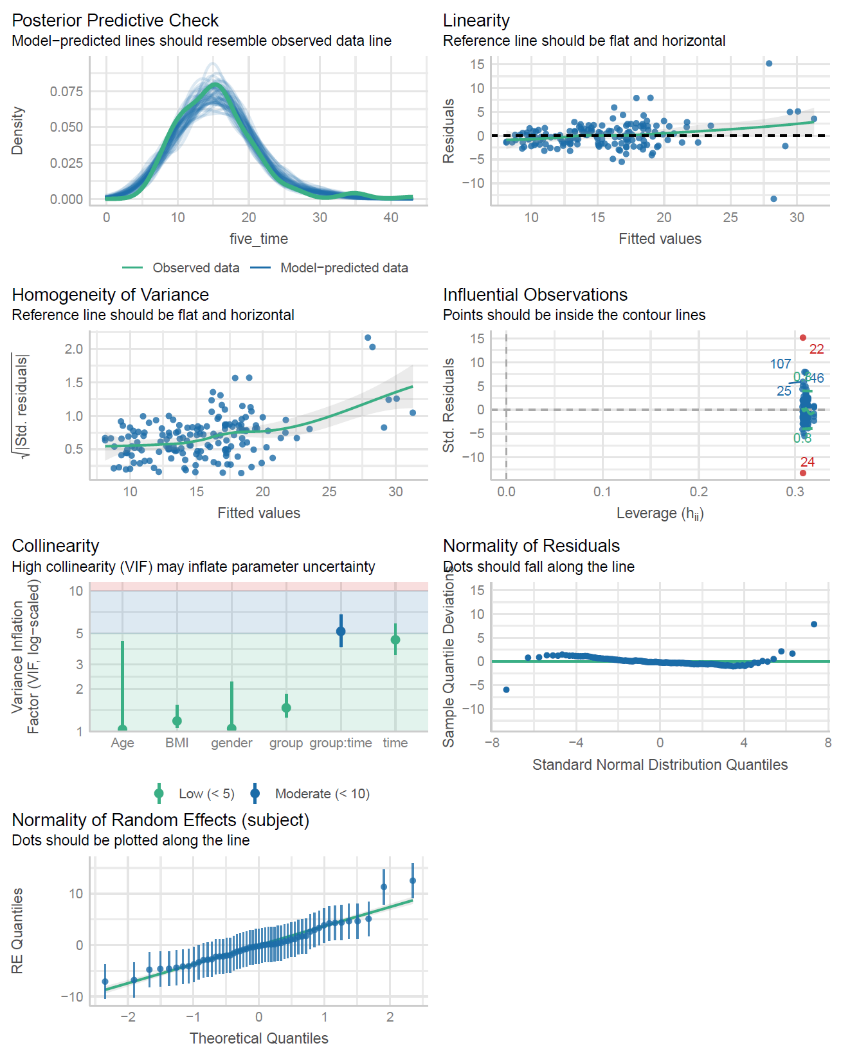

Supplement: Supplementary file 1 — Data S1: Supplementary Information. [file JCSM-17-e70212-s003.docx]
