# Supplementary material for: The Effects of Soy Protein–Rich Meals on Muscle Health of Older Adults Are Linked to Gut Microbiome Modifications
Source: J Cachexia Sarcopenia Muscle. 2026 Jan 25;17(1):e70212. doi: 10.1002/jcsm.70212 (PMC12833501; doi:10.1002/jcsm.70212)
Supplement: Supplementary file 2 — Table S1: Baseline characteristics of participants included in the faecal sample analysis the full study cohort. Table S2: Total energy and macronutrient intake of the 2 groups. Data is presented as mean +/− SEM. Table S3: Outcomes stratified by sex. Data is presented as mean +/− SEM. Table S4: Baseline characteristics of participants included vs. not included from the faecal sample analysis. [file JCSM-17-e70212-s002.docx]

**Supplementary Material**

**Detailed Methods**

**Bioinformatics processing**

From the raw metagenomics sequencing data, low-quality reads were discarded by the sequencing facility and reads belonging to the human genome were removed by mapping the data to the human reference genome (version NCBI37) using KneadData (v0.12.0)[1] and Bowtie2 (v2.3.5.1)[2]. Microbial taxonomic profiles were generated using MetaPhlAn3 (v3.0.14). Microbial general pathways were determined using HUMAnN3 (v3.6.1)[1], which maps DNA/RNA reads to a customized database of functionally annotated pangenomes. HUMAnN3 reported the abundances of gene families from the UniProt Reference Clusters (UniRef90)[3], which were further mapped to microbial pathways from the MetaCyc metabolic pathway database[4].

**Microbiome Phenotype Prediction**

Functional prediction of the gut microbiota was performed using BugBase[5] based on metagenomic sequencing data, a computational tool that predicts organism-level microbiome phenotype. Prior to BugBase analysis, metagenomic reads were processed using SHOGUN (v1.0.8) [6]with the Integrated Microbial Genomes (IMG) database as reference for OTU picking and taxonomic assignment. The tool was used to predict the relative abundance of microorganisms with specific phenotypic traits, including gram staining characteristics and oxygen tolerance (aerobic and anaerobic capabilities). Predictions were made using the default threshold of 0.5 for binary phenotype classification.

**References**

1. Beghini, F., et al., *Integrating taxonomic, functional, and strain-level profiling of diverse microbial communities with bioBakery 3.* ELife, 2021. **10**.

2. Langmead, B., et al., *Scaling read aligners to hundreds of threads on general-purpose processors.* Bioinformatics (Oxford, England), 2019. **35**(3): p. 421-432.

3. *UniProt: a hub for protein information.* Nucleic Acids Research, 2015. **43**(Database issue): p. D204-D212.

4. Caspi, R., et al., *The MetaCyc database of metabolic pathways and enzymes - a 2019 update.* Nucleic Acids Research, 2020. **48**(D1): p. D445-D453.

5. Ward, T., et al., *BugBase predicts organism-level microbiome phenotypes*. 2017, Cold Spring Harbor Laboratory.

6. Hillmann, B., et al., *SHOGUN: Accurate, scalable metagenomic quantification with shallow shotgun sequencing*. 2020, Zenodo.

**Supplementary Tables**

**Supplementary Table 1. Baseline characteristics of participants included in the fecal sample analysis the full study cohort.**

| **Clinical Characteristics** | Full Study Cohort (n = 84) | Sub Cohort (n = 53) | *p*-value |
| --- | --- | --- | --- |
| **General Information** mean (SD) | | | |
| Sex F (%) | 52 (61.9) | 33 (62.3) | 1 |
| Age | 84.89 (6.97) | 84.68 (6.76) | 0.86 |
| **Anthropometric Parameters** Mean (SD) | | | |
| Height | 1.56 (0.09) | 1.57 (0.08) | 0.434 |
| Weight | 57.66 (10.23) | 57.77 (10.05) | 0.954 |
| BMI | 23.72 (3.42) | 23.40 (3.23) | 0.582 |
| **Muscle Function Index** Mean (SD) | | | |
| CC (cm) | 33.22 (2.98) | 33.39 (3.11) | 0.741 |
| SMI (**kg/m^2^**) | 6.14 (1.00) | 6.11 (0.98) | 0.847 |
| HGS (kg) | 19.36 (5.89) | 19.09 (5.40) | 0.789 |
| 6-meter walk (s) | 12.10 (5.29) | 10.73 (5.43) | 0.146 |
| 5-time chair stand (s) | 17.34 (5.18) | 16.47 (5.82) | 0.364 |
| SPPB | 6.43 (2.94) | 7.55 (2.79) | **0.029** |
| **Blood Biomarkers** Mean (SD) | | | |
| IL-6 (pg/ml) | 7.14 (7.43) | 7.19 (8.74) | 0.972 |
| IGF-1 (ng/ml) | 92.61 (25.34) | 90.94 (28.23) | 0.721 |
| Hs-CRP (mg/L) | 1.96 (2.27) | 1.80 (2.15) | 0.676 |
| 25-OH-VD (μg/L) | 17.27 (7.02) | 17.09 (6.86) | 0.88 |

Abbreviations: CC, calf circumference; SMI, skeletal muscle index; HGS, handgrip strength; SPPB, short physical performance battery; IL-6, Interleukin-6; IGF-1, Insulin-like Growth Factor 1; Hs-CRP, High-sensitivity C-reactive Protein; 25-OH-VD, 25-Hydroxyvitamin D.

**Supplementary Table 2. Total energy and macronutrient intake of the 2 groups. Data is presented as mean +/- SEM.**

|  | **Control** | | | **Intervention** | | | ***p^b^*** |
| --- | --- | --- | --- | --- | --- | --- | --- |
|  | **W0** | **W12** | ***P^a^*** | **W0** | **W12** | ***p^a^*** |  |
| Energy, kcal | 1399.00 (34.40) | 1462.61 (57.18) | 0.29 | 1443.08 (49.70) | 1402.84 (61.78) | 0.52 | 0.23 |
| Total protein, g | 65.75 (2.09) | 64.05 (2.28) | 0.56 | 64.68 (2.34) | 73.10 (2.61) | 0.004 | **0.01** |
| Animal protein, g | 34.48 (1.75) | 36.89 (1.54) | 0.31 | 35.80 (1.36) | 23.25 (1.35) | <0.001 | **<0.001** |
| Soy protein, g | 3.65 (0.61) | 2.00 (0.13) | 0.01 | 4.21 (0.49) | 27.44  (0.86) | <0.001 | **<0.001** |
| Plant protein, g | 27.61 (1.77) | 25.21 (1.17) | 0.25 | 24.69 (1.60) | 22.43 (1.11) | 0.17 | 0.96 |
| Fat, g | 52.22 (2.33) | 48.52 (2.13) | 0.16 | 56.49 (2.79) | 44.82 (2.41) | <0.001 | 0.04 |
| Carbohydrates, g | 166.51 (5.82) | 192.42 (8.96) | 0.01 | 168.96 (5.69) | 176.76 (8.77) | 0.35 | 0.16 |

*p^a^* represents the within-group change from baseline to the end of the intervention after 12 weeks. *p^b^* is the time×group difference p value, calculated using a linear mixed-effects model.

**Supplementary Table 3. Outcomes stratified by sex. Data is presented as mean +/- SEM**

|  | **Mean (SEM)** | | | **Mean (95% CI)** | ***p* Value** | |
| --- | --- | --- | --- | --- | --- | --- |
| **Variables** | **Week 0** | **Week 6** | **Week 12** | **Change from baseline** | **Time** | **Interaction** |
| **Women** | | | | | | |
| **CC, cm** | | | | | | |
| Intervention | 33.950 (0.48) | 34.31 (0.53) | 34.37 (0.51) | 0.42 (-0.12, 0.96) | 0.12 | **0.01** |
| Control | 31.40 (0.87) | 31.80 (0.78) | 30.96 (0.88) | -0.45 (-0.91, 0.02) | 0.06 |  |
| **SMI, kg/m^2^** |  | | | | | |
| Intervention | 5.82 (0.10) | 5.95 (0.20) | 5.92 (0.14) | 0.10 (-0.10, 0.28) | 0.61 | 0.70 |
| Control | 5.32 (0.19) | 5.36 (0.19) | 11.16 (1.23) | 0.17 (-0.06, 0.40) | 0.11 |  |
| **6-metre walk, s** | | | | | | |
| Intervention | 10.55 (1.40) | 10.25 (1.27) | 10.84 (1.09) | 0.29 (-1.38, 1.96) | 0.69 | 0.49 |
| Control | 10.14 (1.22) | 10.50 (1.31) | 11.15 (1.23) | 1.00 (-0.33, 2.35) | 0.18 |  |
| **HGS, kg** | | | | | | |
| Intervention | 17.17 (0.84) | 18.25 (0.75) | 19.11 (0.84) | 1.93 (0.46, 3.40) | **0.01** | 0.37 |
| Control | 16.31 (0.98) | 17.5 (0.98) | 17.19 (1.16) | 0.87 (-1.00, 2.75) | 0.34 |  |
| **5-time chair stand, s** | | | | | | |
| Intervention | 15.20 (0.90) | 15.19 (0.84) | 14.44 (1.31) | -0.76 (-2.71, 1.20) | 0.39 | 0.47 |
| Control | 17.57 (1.67) | 14.71 (0.93) | 15.46 (2.03) | -2.11 (-4.93, 0.71) | 0.20 |  |
| **SPPB** | | | | | | |
| Intervention | 7.63 (0.67) | 7.81 (0.75) | 8.25 (0.76) | 0.63 (-0.02, 1.27) | 0.12 | 0.62 |
| Control | 7.71 (0.65) | 8.24 (0.65) | 8.00 (0.77) | 0.29 (-0.98, 1.57) | 0.58 |  |
| **Men** | | | | | | |
| **CC, cm** | | | | | | |
| Intervention | 35.22 (0.45) | 35.86 (0.50) | 36.02 (0.50) | 0.35 (0.10, 1.50) | **0.03** | **< 0.001** |
| Control | 34.15 (1.02) | 34.36 (0.84) | 32.53 (0.76) | -1.62 (-2.56, -0.69) | **< 0.001** |  |
| **SMI, kg/m^2^** | | | | | | |
| Intervention | 7.04 (0.17) | 7.38 (0.18) | 7.12 (0.12) | 0.08 (-0.08, 0.23) | 0.71 | 0.45 |
| Control | 6.98 (0.27) | 6.88 (0.26) | 6.89 (0.25) | -0.08 (-0.20, 0.04) | 0.35 |  |
| **6-metre walk, s** | | | | | | |
| Intervention | 11.03 (2.29) | 9.06 (1.33) | 9.21 (1.26) | -1.82 (-4.14, 0.51) | 0.14 | **0.02** |
| Control | 11.65 (1.55) | 11.83 (1.57) | 13.00 (1.54) | 1.36 (0.23, 2.48) | 0.09 |  |
| **HGS, kg** | | | | | | |
| Intervention | 23.64 (1.41) | 25.57 (1.03) | 23.97 (1.27) | 0.33 (-2.99, 3.65) | 0.81 | 0.67 |
| Control | 22.45 (2.00) | 23.71 (2.22) | 23.51 (1.77) | 1.06 (-0.81, 2.92) | 0.34 |  |
| **5-time chair stand, s** | | | | | | |
| Intervention | 13.90 (1.43) | 12.98 (1.37) | 10.91 (1.28) | -2.99 (-4.38, -1.59) | **< 0.001** | 0.56 |
| Control | 18.75 (2.12) | 18.54 (1.93) | 13.50 (1.54) | -2.25 (-4.18, -0.31) | 0.03 |  |
| **SPPB** | | | | | | |
| Intervention | 8.44 (0.99) | 9.33 (0.78) | 10.00 (0.67) | 1.56 (0.68, 2.43) | 0.001 | 0.40 |
| Control | 6.45 (0.92) | 7.55 (0.65) | 7.45 (0.68) | 1.00 (-0.12, 2.12) | 0.06 |  |

*P_(Time)_* represents the within-group change from baseline to the end of the intervention after 12 weeks. *p_(Interaction)_* is the time×group difference p value, calculated using a linear mixed-effects model. Abbreviations: CC, calf circumference; SMI, skeletal muscle index; HGS, handgrip strength; SPPB, short physical performance battery.

**Supplementary Table 4. Baseline characteristics of participants included vs. not included from the fecal sample analysis**

| **Clinical Characteristics** | Included (n=53) | Not Included (n=31) | *p*-value |
| --- | --- | --- | --- |
| **General Information** Mean (SD) | | | |
| Sex F (%) | 33 (62.3) | 19 (61.3) | 1 |
| Age | 84.68 (6.76) | 85.26 (7.41) | 0.716 |
| **Anthropometric Parameters** Mean (SD) | | | |
| Height (m) | 1.57 (0.08) | 1.54 (0.10) | 0.112 |
| Weight (kg) | 57.77 (10.05) | 57.49 (10.69) | 0.904 |
| BMI | 23.40 (3.23) | 24.28 (3.72) | 0.258 |
| **Muscle Function Index** Mean (SD) | | | |
| CC (cm) | 33.39 (3.11) | 32.91 (2.77) | 0.482 |
| SMI (**kg/m^2^**) | 6.12 (0.97) | 6.19 (1.07) | 0.732 |
| HGS (kg) | 19.09 (5.40) | 19.82 (6.72) | 0.588 |
| 6-meter walk (s) | 10.73 (5.43) | 14.44 (4.17) | **0.002** |
| 5-time chair stand (s) | 16.47 (5.82) | 18.83 (3.45) | **0.044** |
| SPPB | 7.55 (2.79) | 4.52 (2.11) | **<0.001** |
| **Blood Biomarkers** Mean (SD) | | | |
| IL-6 (pg/ml) | 7.19 (8.74) | 7.05 (4.52) | 0.937 |
| IGF-1 (ng/ml) | 90.94 (28.23) | 95.45 (19.54) | 0.434 |
| Hs-CRP (mg/L) | 1.80 (2.15) | 2.24 (2.47) | 0.391 |
| 25-OH-VD (μg/L) | 17.09 (6.86) | 17.59 (7.40) | 0.755 |

Abbreviations: CC, calf circumference; SMI, skeletal muscle index; HGS, handgrip strength; SPPB, short physical performance battery; IL-6, Interleukin-6; IGF-1, Insulin-like Growth Factor 1; Hs-CRP, High-sensitivity C-reactive Protein; 25-OH-VD, 25-Hydroxyvitamin D.

**Abbreviations list**

| CC | Calf Circumference |
| --- | --- |
| SMI | Skeletal Muscle Index |
| HGS | Handgrip strength |
| SPPB | Short Physical Performance Battery |
| IL-6 | Interleukin-6 |
| IGF-1 | Insulin-like Growth Factor 1 |
| Hs-CRP | High-sensitivity C-reactive Protein |
| 25-OH-VD | 25-Hydroxyvitamin D |
