# Supplementary material for: The Effects of Soy Protein–Rich Meals on Muscle Health of Older Adults Are Linked to Gut Microbiome Modifications
Source: J Cachexia Sarcopenia Muscle. 2026 Jan 25;17(1):e70212. doi: 10.1002/jcsm.70212 (PMC12833501; doi:10.1002/jcsm.70212)
Supplement: Supplementary file 3 — Figure S1: Changes in muscle health outcomes and blood biomarkers A‐C: Changes from baseline in (A) SPPB, (B)HGS, (C) 5‐time chair stand test. Data presented as mean ± standard error. *p (Time) < 0.05, **p (Time) < 0.01 denote p‐adjusted values for the time coefficient within the same group. D‐F: Paired boxplot shows the changes in (D) 25‐OH‐VD, (E) hsCRP and (F) IGF‐1 for each subject. The line inside each box is the median, and the box edges show the range where the middle 50% of data lies. Whiskers extend to the smallest and largest values within 1.5 times the interquartile range. Outliers are shown as individual points. Asterisks indicate significant differences. Figure S2: Changes in overall structure of gut microbiome A: Principal coordinate analysis of Bray‐Curtis distances. The axes are labelled with the percent variance explained. B: Paired boxplot shows the changes in Shannon index over time for each subject in both groups. Figure S3: Changes in gut microbiota genus and species Lineplots show the temporal changes in specific genus and species. Data presented as mean ± < 0.01 denote p values for the time×group interaction coefficient in the LMM model.*p (Time) < 0.05, **p (Time) < 0.01 denote p values for the time coefficient within the same group. Figure S4: Changes in specefic species < 0.05, ##p (Time×group) < 0.01 denote p values for the time×group interaction coefficient in the LMM model.*p (Time) < 0.05, **p (Time) < 0.01 denote p values for the time coefficient within the same group. “CC_d” and “CC_u” indicate participants whose calf circumference decreased/remained stab le or increased, respectively; “SMI_d” and “SMI_u” indicate participants whose SMI decreased/rema ined stable or increased, respectively. Figure S5: Changes in faecal SCFAs A‐F: Paired boxplot shows the changes in six SCFAs for each subject. The line inside each box is the median, and the box edges show the range where the middle 50% of data lies. Whiskers extend to the smallest and [file JCSM-17-e70212-s001.pdf]

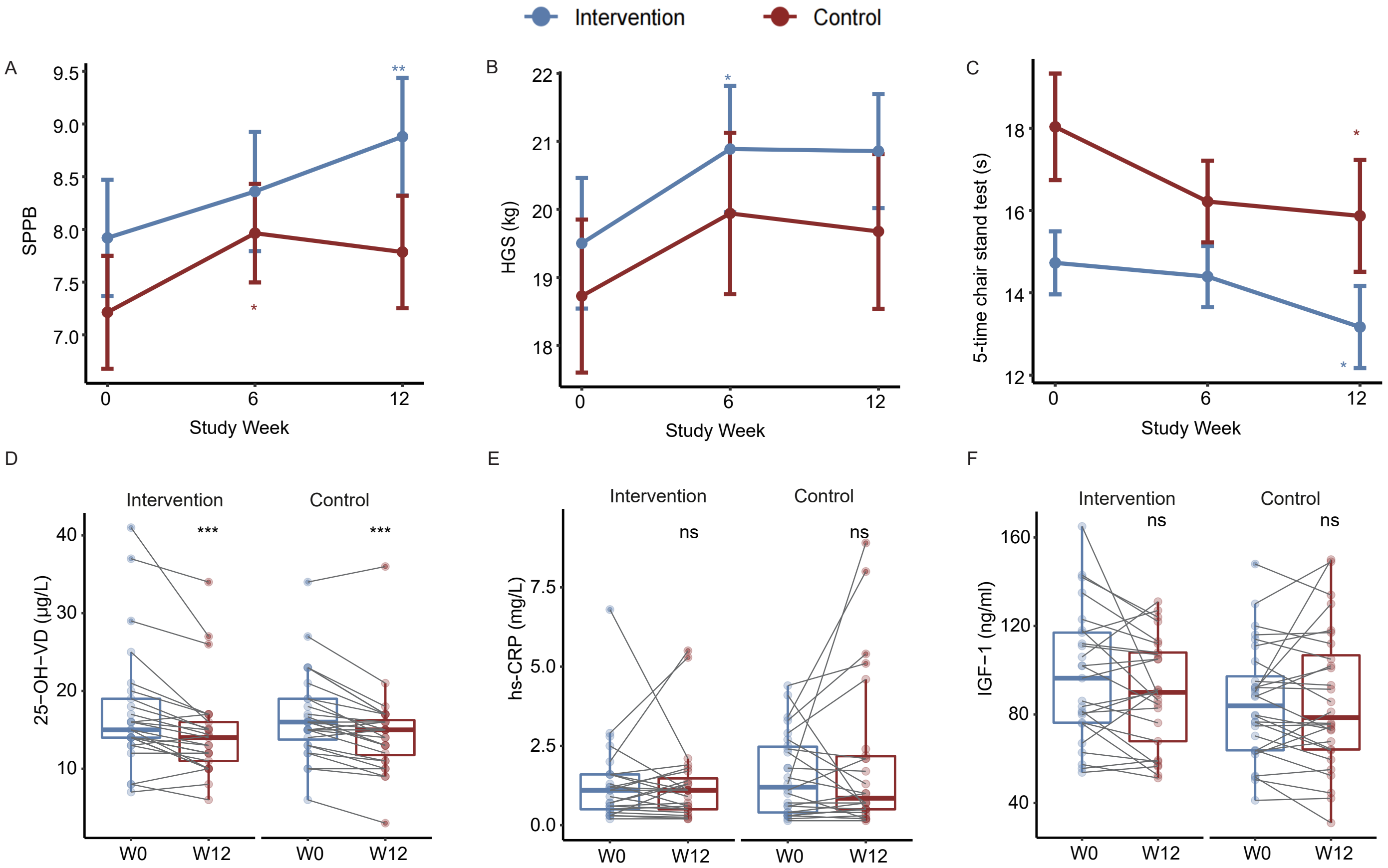

Figure S1. Changes in muscle health outcomes and blood biomarkers

A-C: Changes from baseline in (A) SPPB, (B)HGS, (C) 5-time chair stand test. Data presented as mean ± standard error. $*p_{(Time)} < 0.05$, $**p_{(Time)} < 0.01$ denote p-adjusted values for the time coefficient within the same group.

D-F: Paired boxplot shows the changes in (D) 25-OH-VD, (E) hsCRP and (F) IGF-1 for each subject. The line inside each box is the median, and the box edges show the range where the middle 50% of data lies. Whiskers extend to the smallest and largest values within 1.5 times the interquartile range. Outliers are shown as individual points. Asterisks indicate significant differences.

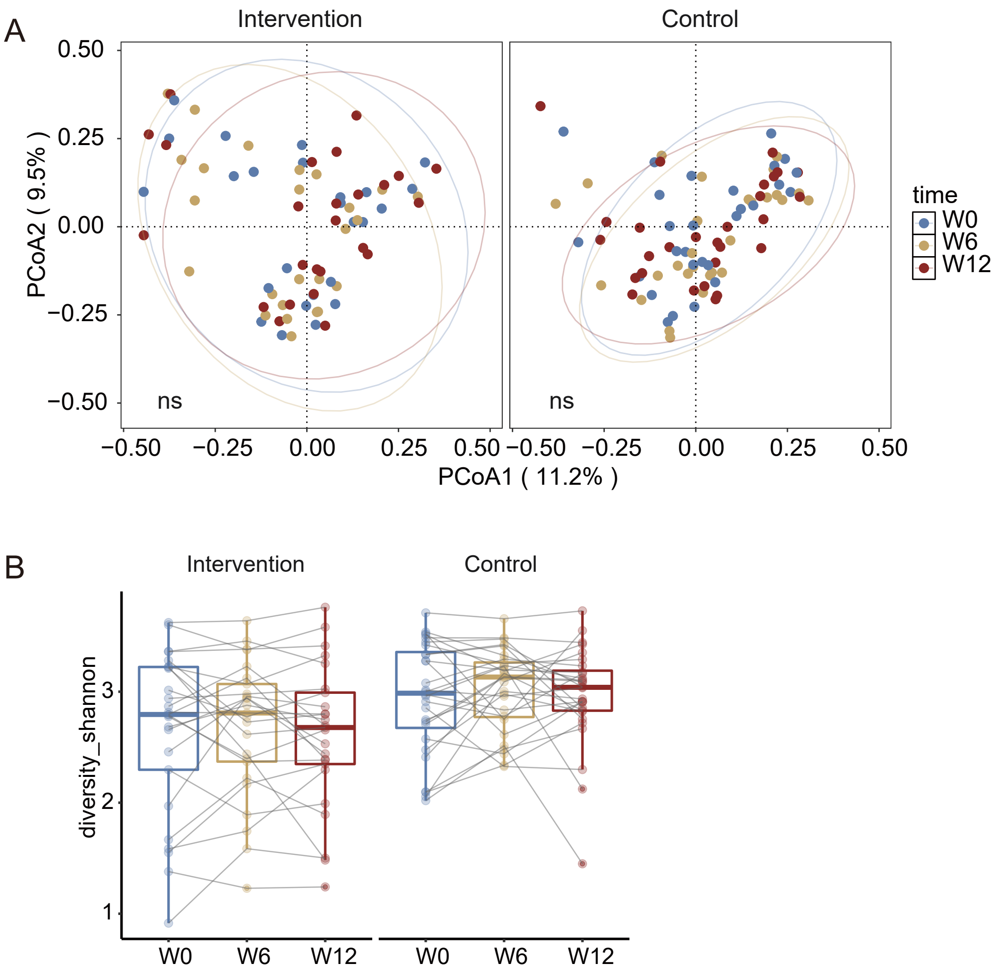

Figure S2. Changes in overall structure of gut microbiome

A: Principal coordinate analysis of Bray-Curtis distances. The axes are labelled with the percent variance explained.

B: Paired boxplot shows the changes in Shannon index over time for each subject in both groups.

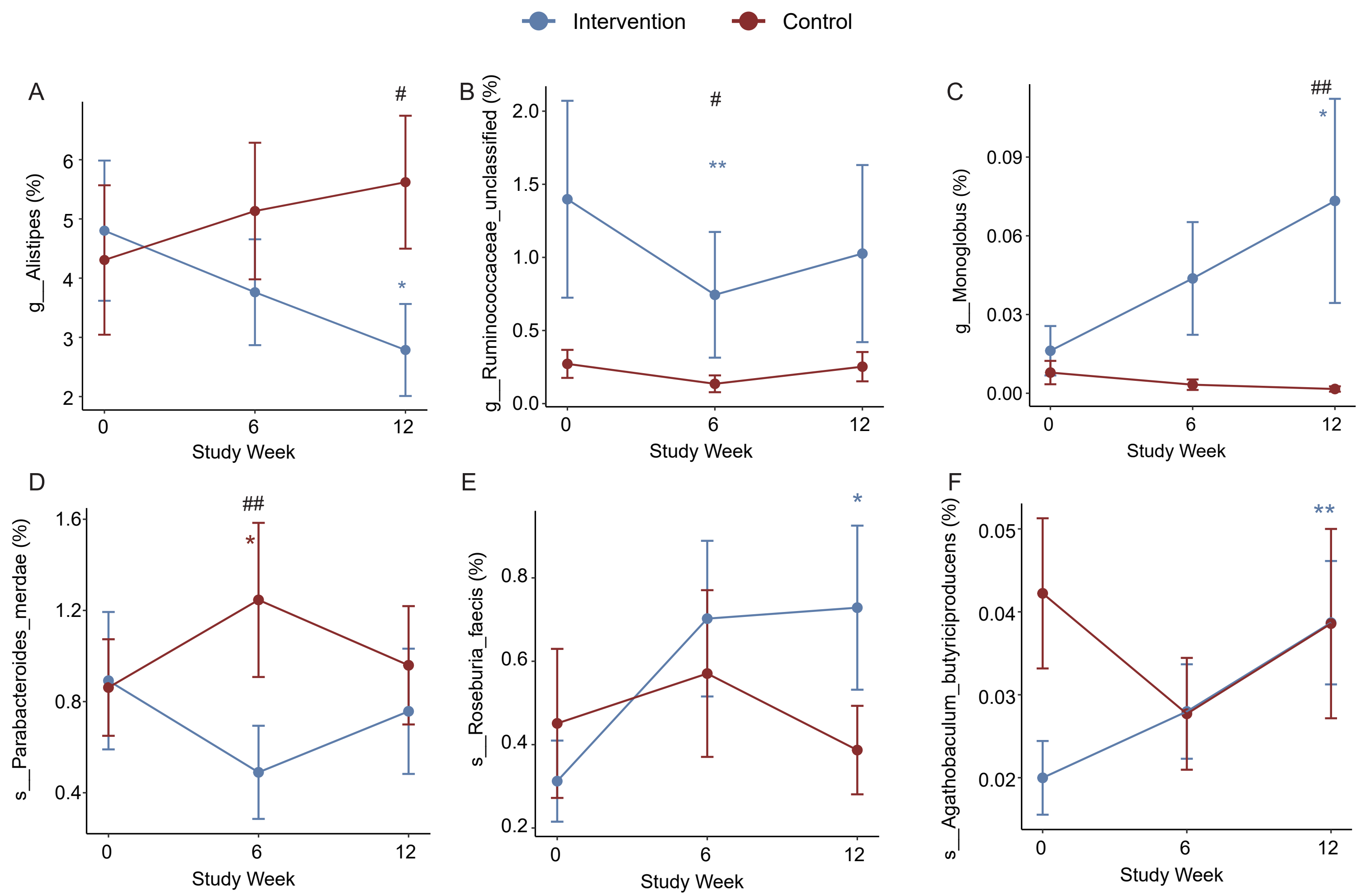

Figure S3. Changes in gut microbiota genus and species

Lineplots show the temporal changes in specific genus and species. Data presented as mean ± standard error. #$p_{(\text{Time×group})} < 0.05$, ##$p_{(\text{Time×group})} < 0.01$ denote p values for the time×group interaction coefficient in the LMM model .*$p_{(\text{Time})} < 0.05$, **$p_{(\text{Time})} < 0.01$ denote p values for the time coefficient within the same group.

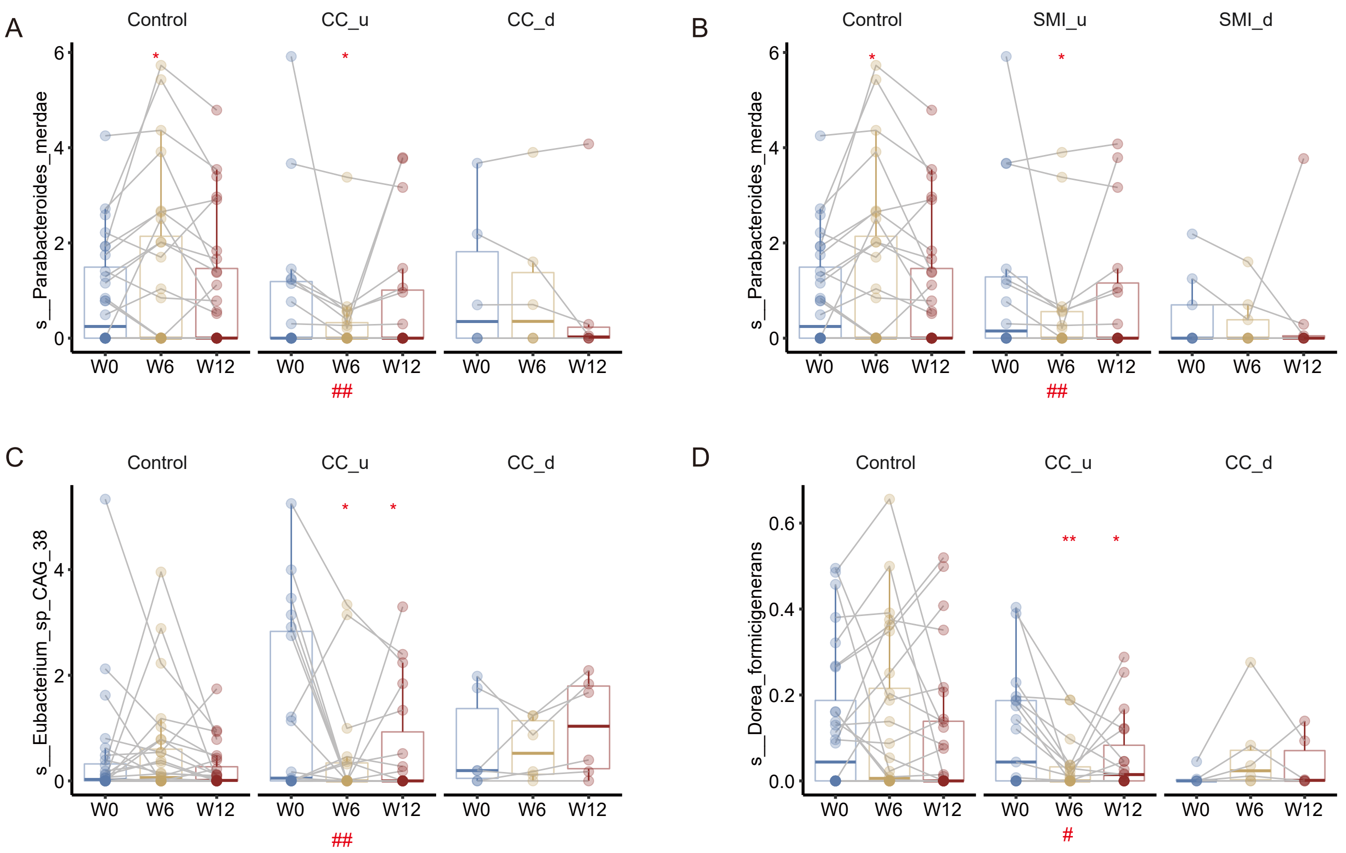

Figure S4. Changes in specefic species

A-D: Paired boxplot shows the changes in three species for each subject. #$p_{(\text{Time×group})}$ < 0.05, ##$p_{(\text{Time×group})}$ < 0.01 denote p values for the time×group interaction coefficient in the LMM model .*$p_{(\text{Time})}$ < 0.05, **$p_{(\text{Time})}$ < 0.01 denote p values for the time coefficient within the same group. "CC_d" and "CC_u" indicate participants whose calf circumference decreased/remained stab le or increased, respectively; "SMI_d" and "SMI_u" indicate participants whose SMI decreased/rema ined stable or increased, respectively.

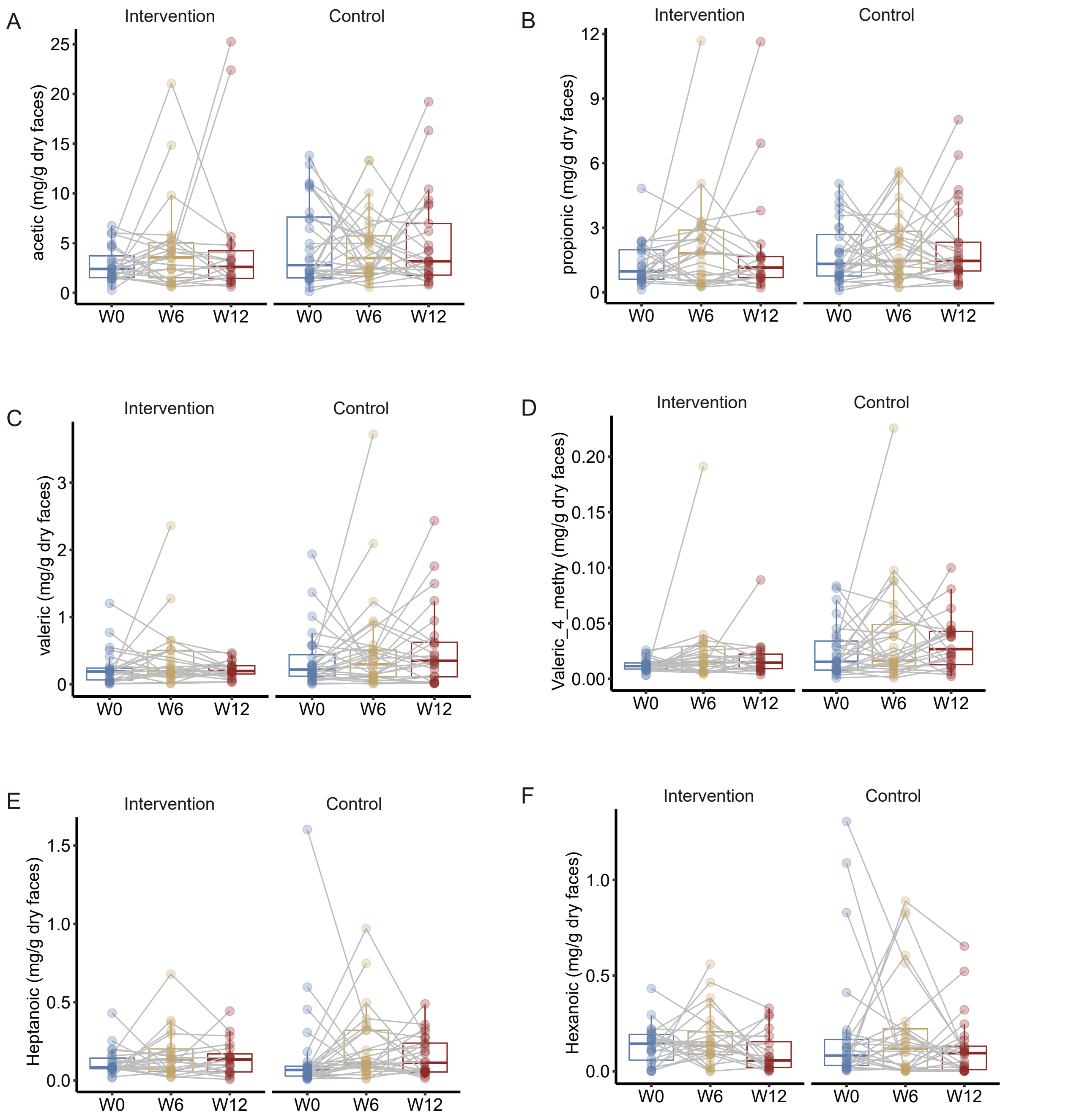

Figure S5. Changes in fecal SCFAs

A-F: Paired boxplot shows the changes in six SCFAs for each subject. The line inside each box is the median, and the box edges show the range where the middle 50% of data lies. Whiskers extend to the smallest and largest values within 1.5 times the interquartile range. Outliers are shown as individual points.

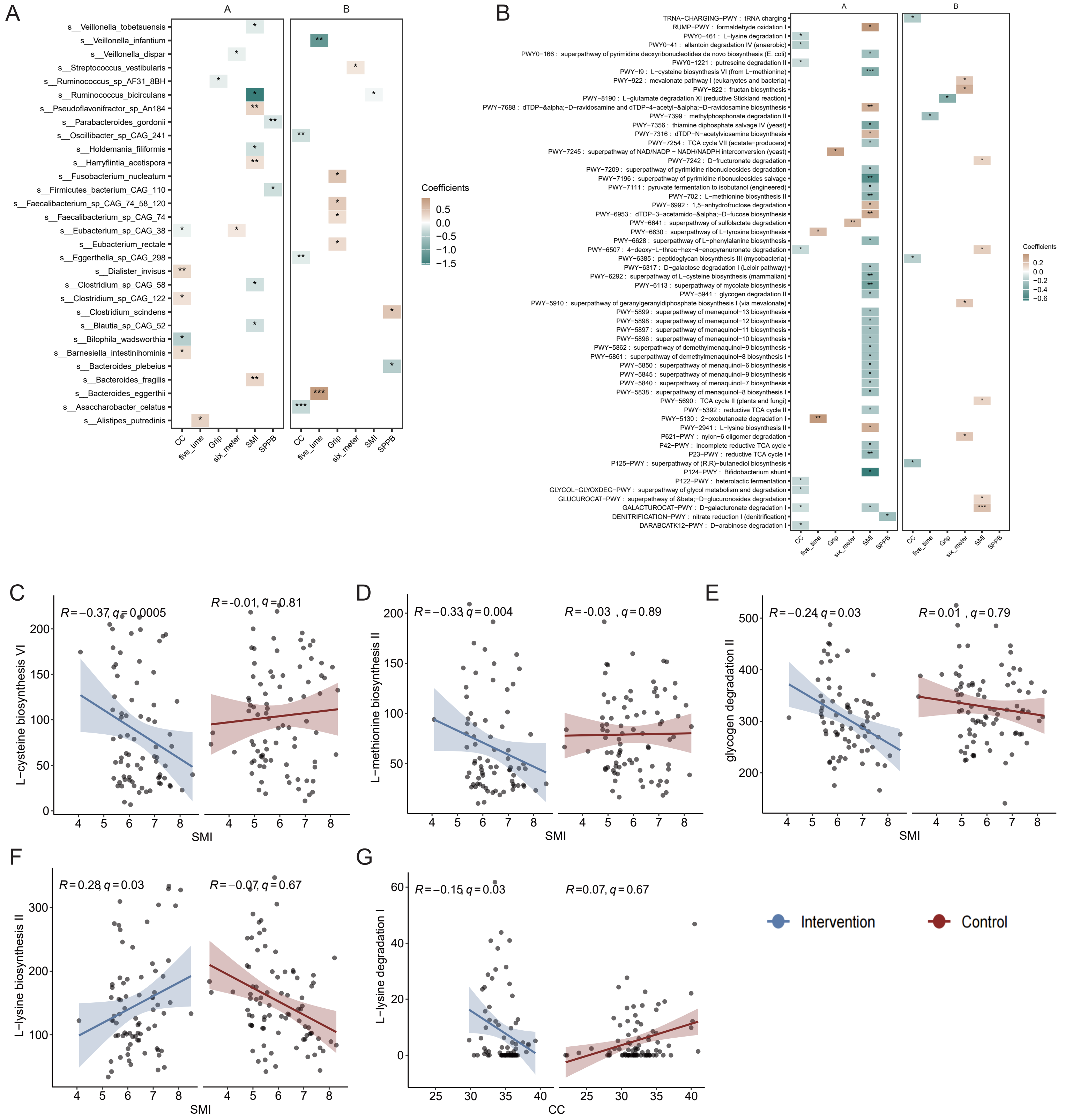

Figure S6. Associations between gut microbiota and muscle health outcomes

A-B: Heatmap of p values and coefficients of associations between species (A) or payhways (B) and muscle health outcomes across the whole study period. Only correlations with p-values < 0.05 for time*group terms in the LMM model are shown. *q < 0.05; **q< 0.01; ***q < 0.001 gives the p-adjusted values for the variable association coefficient in the LMM model performed separately in two groups.

C-G: Scatter plots displaying correlations between metabolic pathway activity and muscle function measurements in intervention (blue) and control (red) groups. (C-D) Sulfur-containing amino acid biosynthesis pathways (L-cysteine and L-methionine) negatively correlate with SMI in the intervention group. (E) Glycogen degradation pathway negatively correlates with SMI in the intervention group (R=-0.24, q=0.03). (F) L-lysine biosynthesis positively associates with SMI in the intervention group (R=0.28, q=0.03). (G) L-lysine degradation pathway negatively correlates with CC in the intervention group (R=-0.15, q=0.03). The smooth curve represents the trend line fitted using Linear Model, and the shaded area indicates the 95% Confidence Interval. The R values and q values displayed in the figure were calculated based on Linear Mixed effects Models (LMM) performed separately within each group.
